# Intelligent Pyroptosis Inducer for Precise and Augmented Tumor Therapy Through Specific Activation Pyroptosis in Tumor

**DOI:** 10.1002/advs.202407713

**Published:** 2024-11-27

**Authors:** Linlin Huo, Shiqi Zhu, Muyao Li, Mingya Tan, Mengke Fan, Jiayi Zhao, Jie Zeng, Meiling Liu, Kunyan Liu, Chao Tong, Zhenghuan Zhao

**Affiliations:** ^1^ College of Basic Medical Sciences Chongqing Medical University Chongqing 400016 China; ^2^ College of Life Sciences and Medicine Chengdu University of Traditional Chinese Medicine Chengdu 610075 China; ^3^ National Clinical Research Center for Child Health and Disorders Ministry of Education Key Laboratory of Child Development and Disorders Children's Hospital of Chongqing Medical University Chongqing 401122 China

**Keywords:** H_2_O_2_ responsive, precise tumor therapy, ROS scavenger/generator, specific pyroptosis

## Abstract

Pyroptosis inducer, a powerful anti‐tumor agent that causes obvious programmed cell death and immune stimulation, has been challenged to trigger specific pyroptotic tumor cell death while keeping pyroptosis silence in normal cells. Here, an intelligent inducer is reported that acts as a reactive oxygen species (ROS) scavenger in normal cells to keep pyroptosis silence, while serving as ROS generator to induce obvious pyroptotic tumor cells death dependent on high hydrogen peroxide levels and near‐infrared laser irradiation. This switchable activity ensures this inducer to precisely kill the tumor cells with augmented immunogenicity while causing minimal damage to normal cells. Moreover, the catalase‐like activity endows this inducer to overcome limitation of tumor hypoxia on ROS generation and show significant pyroptosis activation, further initiating the immune response to inhibit the tumor metastases in vivo. This study provides valuable insights into design new pyroptosis inducer with controllable pyroptosis activity to specifically induce programmed tumor cell pyroptosis for precise and augmented tumor therapy with minimal side effects.

## Graphical abstract

The hydrogen peroxide responsive specific pyroptosis inducer triggers specific pyroptotic tumor cell death and maintains pyroptosis silence through differentially controlling reactive oxygen species levels in tumor and normal cells, precisely inhibiting the growth of primary and metastatic tumors with negligible side effect.

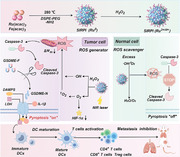

## 1 Introduction

Tumor immunotherapy, which enhances the natural immune system to defense the tumor cells, has shown a prospective future in tumor inhibition.^[^ ^1^ ^]^ However, the therapeutic efficiency of immunotherapy in solid tumors has been severely challenged by the low immunogenicity of tumor microenvironment (TME).^[^ ^2^ ^]^ This limitation emphasizes the urgent need to ameliorate TME and improve the immunogenicity for effective tumor therapy. Pyroptosis, as a mode of programmed cell death, is distinguished from apoptosis and necrosis by the distinctive features of cell swelling, membrane rupture, bubble‐like protrusions appear, pro‐inflammatory cytokines, and cellular contents release.^[^ ^3^ ^]^ It is an effective mode to enhance tumor immunogenicity and initiate immune response. For the typical pathway of pyroptosis, it is primarily induced by the stimulation of bacteria, viruses, toxins, or chemotherapeutic drugs from external or intracellular, and is associated with the recruitment and activation of caspase‐1. Specifically, the gasdermin D (GSDMD) is cleaved by activated caspase‐1 to produce GSDMD‐N domain to generate pore in the plasma membrane with size of about 1 µm, allowing the release of inflammatory cytokines like IL‐1β and IL‐18.^[^ ^4^ ^]^ These phenomena result in the strong inflammation and massive exposure of antigen epitopes to augment immunogenicity. Hence, pyroptosis inducers provide innovative study guideline to activate pyroptotic tumor cell death, synergistically increasing tumor immunogenicity and enhancing tumor therapy efficiency.^[^ ^5^ ^]^

Growing evidence has revealed that reactive oxygen species (ROS), as a pyroptosis inducer, initiates pyroptosis for effective tumor therapy via the csapase‐3/GSDME pathway.^[^ ^6^ ^]^ ROS accumulation induces Bax recruitment in the mitochondrial membrane and release cytochrome *c* (cyt *c*).^[^ ^3^, ^7^ ^]^ The released cyt *c* activates caspase‐3 to cleave GSDME and leads to the release of massive inflammatory cytokines and damage‐associated molecular patterns (DAMPs), triggering an immune response and suppressing tumor growth.^[^ ^8^ ^]^ To minimize the damage in normal tissues caused by the pyroptosis, the activatable photosensitizers with the remarkable capacity to specifically increase the ROS levels in tumor and induce pyroptotic tumor cell death have been extensively studied.^[^ ^9^ ^]^ These intelligent pyroptosis inducers show assembled state in normal tissue, resulting in the “off” state on triggering pyroptosis.^[^ ^10^ ^]^Alternatively, these inducers respond to TME, e.g., acidic environment or high glutathione (GSH) level, and disassemble to achieve “on” state on triggering pyroptosis.^[^ ^11^ ^]^ These novel studies provide insights into the design of specific pyroptosis inducers with minimal side effects. Unfortunately, pragmatic application of these activatable strategies is critically limited by the ineluctable inflammation response of normal tissue, which is characterized by oxidative stress evoked by elevated ROS level caused by the released inflammatory factor from dead tumor cells.^[^ ^12^ ^]^ Besides, TME with hypoxia nature, which imparts resistance to ROS generation, resulting the inefficient activation of pyroptosis and tumor therapeutic efficacy with the low immunogenicity.^[^ ^13^ ^]^ Thus, it is desirable to develop an intelligent and highly efficient pyroptosis inducer that acts as ROS scavenger in normal tissue to keep pyroptosis silence for normal cells protection, while serving as an efficient ROS generator with TME regulation capacity to trigger significant pyroptosis and synergistically increase tumor immunogenicity for robust tumor therapy.

**Scheme 1:**
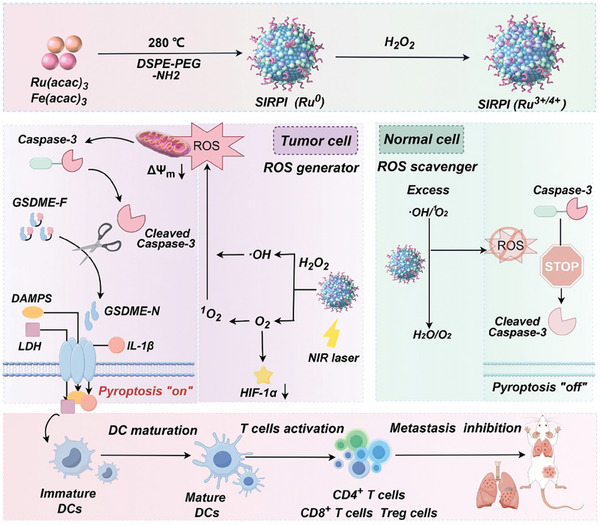
Schematic diagram of SIRPI act as ROS scavenger/generator to induce specific pyroptosis for enhancing immune response. The mechanism of SIRPI, which is responds with H_2_O_2_ to act as ROS scavenger/generator, to augment the immune response and accurately suppress the tumors by inducing specific pyroptotic tumor cell death via the caspase‐3/GSDME pathway.

Herein, we develop a TME responsive specific iron ruthenium pyroptosis inducer (SIRPI) that acts as a highly specific ROS generator to effectively initiate the tumor cell pyroptosis while serving as a ROS scavenger to maintain the pyroptosis related protein in low levels. Specifically, the SIRPI exhibits reducibility under physiological conditions to effectively scavenge the excess ROS in normal tissues, resulting in negligible pyroptosis and toxicity. Whereas, the SIRPI switches from a ROS scavenger to a ROS generator in tumor site owning to the H_2_O_2_ responsive oxidation of Ru and Fe elements. This transformation enables SIRPI to generate amounts of hydroxyl radical (•OH) through oxidated Fe and Ru ions‐based Fenton reactions and singlet oxygen (^1^O_2_) through the free electrons transferring to oxygen under near‐infrared (NIR) laser simulation, ensuring SIRPI induces significant pyroptosis in tumor cells. Additionally, SIRPI relieves the hypoxia of tumor based on its excellent catalase‐like (CAT‐like) enzyme activity, enabling efficient ROS generation to trigger specific pyroptotic tumor cell death via the caspase‐3/GSDME pathway in tumor tissue. The specific tumor pyroptosis leads to the release of inflammatory cytokines and DAMPs into the extracellular, exposing antigenic recognition sites, promoting dendritic cells (DCs) maturation, and amplifying systemic immune responses to inhibit the primary and metastasis lesions. Moreover, the SIRPI with *T*_2_ imaging performance will facilitate real‐time monitoring of tumor progression and therapeutic efficiency (**Scheme** **1**). This specific pyroptosis mediated by SIRPI, which works as vibrant/invalid pyroptosis inducer in tumor/normal tissue, offers a new possibility for the efficient and safety tumor therapy.

## 2 Results and Discussion

### 2.1 Synthesis and Characterization of Specific Iron Ruthenium Pyroptosis Inducer (SIRPI)

**Figure 1:**
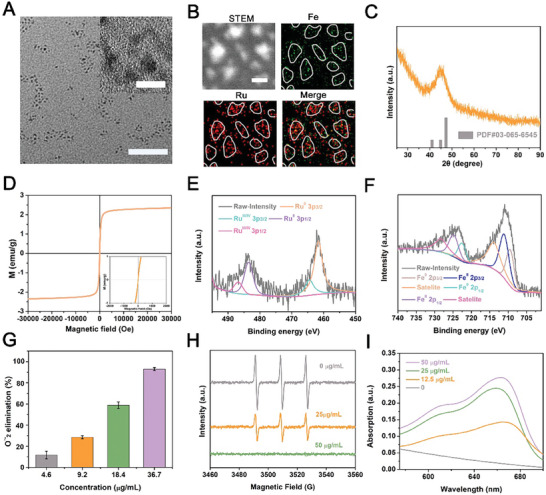
Characterization and reducibility of SIRPI. A) TEM and High‐resolution TEM images of SIRPI, the scale bar is 20 nm and 5 nm, respectively. B) HAADF‐STEM image and elemental mappings of Fe and Ru elements in SIRPI, the scale bar is 2 nm. C) XRD patterns and D) smooth *M–H* curves of SIRPI, indicating the ferromagnetic properties of SIRPI have been successfully synthesized. XPS high‐resolution spectra of E) Ru 3p and F) Fe 2p in SIRPI. G) Elimination of O_2_^·−^ with different concentrations of SIRPI (4.6–36.7 µg mL^−1^). H) ESR spectra of ^1^O_2_ scavenging and I) UV‐vis spectra of •OH with different concentrations of SIRPI (0, 25, 50 µg mL^−1^).

We synthesized the specific iron ruthenium pyroptosis inducer (SIRPI) by thermo‐decomposition of the mixture containing ruthenium acetylacetonate and iron acetylacetonate in 1‐Octadecene. Transmission electron microscopy (TEM) images indicate that the as‐prepared SIRPI exhibit uniform morphology with the diameter of approximately 2.4 ± 0.3 nm (Figure S1, Supporting Information). The uniformly lattice fringes across the entire nanoparticles are clearly observed in high‐resolution TEM (HRTEM) image, affirming the excellent crystallinity of SIRPI (**Figure** **1**A). To further verify the composition of SIRPI, the energy dispersive X‐ray (EDX) elemental mapping was performed. It appears that the iron (Fe) and ruthenium (Ru) signals are homogeneously disperse in the whole nanoparticles, suggesting the simultaneously presence of Fe and Ru element in a SIRPI particle (Figure 1B). Additionally, the X‐ray diffraction (XRD) analysis exhibits the characteristic diffraction peaks of SIRPI match well with iron ruthenium alloy (JCPDS No.03‐065‐6545), which coordinately revealing the basic crystal structure of as‐prepared SIRPI (Figure 1C). The magnetic property of SIRPI was investigated using superconducting quantum interference device (SQUID) magnetometer at room temperature (300 K). The field‐dependent magnetization (*M‐H*) curves show that SIRPI exhibits superparamagnetic behavior without any hysteresis at room temperature, which is expected to be used as a *T*_2_ contrast agent for magnetic resonance imaging (Figure 1D). Subsequently, we transferred SIRPI into aqueous by hydrophilic 1,2‐distearoyl‐sn‐glycero‐3‐phosphoethanolamine‐N‐ [amino (polyethylene glycol)‐2000] (DSPE‐PEG2000 amine). The hydrodynamic size and zeta potential of water‐soluble SIRPI were measured by dynamic light scattering (DLS). It displays that the size and potential of SIRPI are about 10.8 nm and + 3.4 mV, promising the SIRPI with the well potential to remain stable in the circulation (Figure S2, Supporting Information).

### 2.2 Reducibility‐Mediated ROS Scavenging Activity of SIRPI in Physiological Condition

To assess the oxidation‐reduction property of SIRPI, we performed the X‐ray photoelectron spectroscopy (XPS) to analyze Fe and Ru valence states in mimic physiological condition. The XPS peak‐fitting analyses reveal that the ratios of Ru^0^ (at 461.6 eV) to Ru^3+/4+^ (at 464.5 eV) and Fe^0^ (at 709.5 eV) to Fe^2+^ (at 711.1 eV) is about 3.3:1 and 0.6:1 (Figure 1E, F). The large proportion of Ru^0^ imparts SIRPI with the reducibility, which is expected to act as ROS scavenger in the physiological condition^34^. With this hypothesis, we assessed the potential of SIRPI to eliminate superoxide anion (O_2_ **^·^** ^−^), singlet oxygen (^1^O_2_), and hydroxyl radical (**·**OH) in physiological condition, respectively. We first measured the scavenging activity of O_2_ **^·^** ^−^ through adding different concentration of SIRPI (4.6‐36.7 µg mL^−1^) into a mixture of L‐methionine and riboflavin after 15 min ultraviolet light irradiation and used nitrotetrazolium blue chloride (NBT) as probe. It appears that SIRPI shows high O_2_ **^·^** ^–^ scavenging activity in concentration‐dependent manner. The O_2_ **^·^** ^–^ elimination ratio is as high as ≈92.7% when SIRPI concentration reaches to 36.7 µg mL^−1^ (Figure 1G). Subsequently, electron spin resonance (ESR) spectra were applied to detect ^1^O_2_ levels with or without SIRPI using 2,2,6,6‐tetramethylpiperidin‐1‐oxygen (TEMP) as spin trap. Interestingly, the signal intensity of TEMP/^1^O_2_ at the characteristic peaks are effectively reduced by SIRPI in concentration dependence manner. It should note that complete scavenging is observed when the concentration of SIRPI is as low as 50 µg mL^−1^, implying the effective elimination of ^1^O_2_ by SIRPI at physiological environment (Figure 1H). The •OH scavenging activity was investigated by adding SIRPI into the solution containing •OH using methylene blue (MB) as specific probe. Remarkably, we observed gradual recovery of the characteristic absorbance peak of MB with the increase of SIRPI concentration, indicating the effective scavenging of •OH by SIRPI (Figure 1I). These results prove that SIRPI with the reducibility exhibits the superior ROS elimination performance under physiological conditions, which is expected to minimize the ROS induced pyroptosis in normal tissues.

### 2.3 ^1^O_2_ Generation Activity of SIRPI under NIR Laser Irradiation

**Figure 2:**
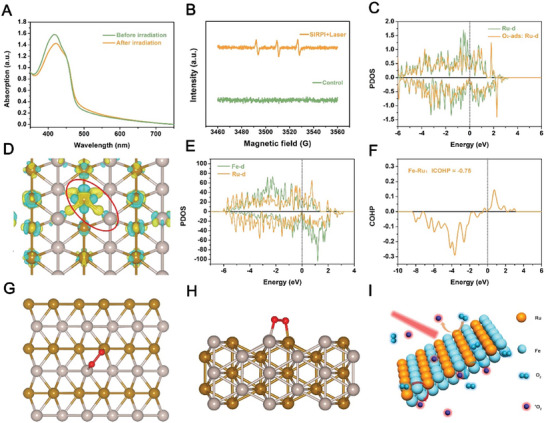
^1^O_2_ generation activity of SIRPI. A) UV‐vis spectra of DPBF and B) ESR spectra of TEMP)/ ^1^O_2_ to detect ^1^O_2_ by SIRPI under NIR laser irradiation. C) The pre‐ and post‐ O_2_ adsorption PDOS for the Ru d orbital. D) Electron deposition between Fe─Ru bond. The public electron pair in the Fe─Ru bond is represented by the red circle, while the yellow color represents the charge increase and the cyan color represents the charge decrease. E) Fe─d and Ru‐d hybridization during O_2_ adsorption. F) The Fe−Ru bond strength analysis using the COHP. G) The O_2_ adsorption structure of SIRPI. H) Simulated structure for ^1^O_2_ generation. I) Schematic illustration of the ^1^O_2_ generation by SIRPI under 808 nm NIR laser stimulation.

Ru‐based complexes have been widely used as photosensitizers to generate ^1^O_2_ and work as potential photodynamic therapy agent.^[^ ^14^ ^]^ To investigate the ^1^O_2_ generation activity of SIRPI, we exposed SIRPI to an 808 nm NIR laser for 10 min and monitored ^1^O_2_ levels using 1,3‐diphenylisobenzofuran (DPBF) as probe. We observed a 9.2% decrease in the DPBF characteristic absorbance peak at 412 nm after NIR laser irradiation (**Figure** **2**A). Consistently, the signal intensity of TEMP/^1^O_2_ in ESR spectra increased with the adding of SIRPI (Figure 2B). These results clearly indicate that SIRPI successfully generates ^1^O_2_ under NIR laser irradiation. However, the signal changes in both DPBF and TEMP/^1^O_2_ are limited compared to the control group, indicating relatively low ^1^O_2_ generation efficiency of SIRPI under NIR laser irradiation in physiological conditions. These phenomena could be ascribed to the partially elimination of generated ^1^O_2_ based on the ROS scavenging activity of SIRPI in physiological condition, which is beneficial to diminish ^1^O_2_ generation in normal tissue in further therapy. To understand the detailed physical and chemical mechanisms of ^1^O_2_ generation by SIRPI under NIR laser irradiation, we performed density functional theory (DFT) analyses.^[^ ^15^ ^]^ The surface energies of low index facets of Fe─Ru nanostructure were first calculated. It appears that (110) facet of iron ruthenium nanostructure shows the lowest surface energy, indicating that (110) facet is more likely to form stable structures and preferentially exposed on the surface of SIRPI. We noticed that the Ru‐d orbital is upshifted after O_2_ adsorption compared to the clean slab in the partial density of state (PDOS) spectra, indicating the interaction between Ru d and O_2_ and suggesting that the ^1^O_2_ would form based on the activated electron transfer to O_2_ under extra energy (Figure 2C). According to the previous research, the shared pairs of electrons of the broken Fe−Ru bond may dominate to the O_2_ activation. We employed the PDOS and crystal orbital Hamilton population (COHP) to analyze the Fe−Ru bond strength as function of O_2_ activation. We noticed obvious hybridization that occurred between the Fe─d and Ru‐d orbitals in PDOS (Figure 2E). These results suggest that the main component in the Fe─Ru bond are Fe─d and Ru‐d. Besides, electron deposition between Fe─Ru bond could be observed, implying the adequate free electron supplying during the breaking of Fe─Ru bond (Figure 2D). For COHP, the Fe─Ru bond interactions are mainly between −9.0 eV and 4.0 eV with the antibonding state above −1.0 eV. These results are close to the Fermi energy level, which finally causes the activation of O atoms. The integral COHP (ICOHP) was employed to estimate the bond strength. In theory, the stronger bond is, the more negative ICOHP would be observed. The Fe─Ru bond strength is calculated to be about –0.75. The low bond strength between Fe and Ru ensures that Fe─Ru bond could be easily broken by the extra energy and provide sufficient free electrons to the adsorbed O_2_ and generate ^1^O_2_ (Figure 2F). Additionally, we noticed that the ESR spectra of SIRPI exhibited a pair of inverted pinnacles (Figure S3, Supporting Information). This result implies the existence of high‐density defects in SIRPI, which endow SIRPI with the efficient exposure of active sites and optimized electronic structures to enhance the ^1^O_2_ yield.^[^ ^16^ ^]^ Overall, the prepared SIRPI effectively transfers free electrons from the broken of Fe─Ru bond to O_2_ and generate ^1^O_2_ under NIR laser irradiation (Figure 2G–I). On the other hand, the ROS scavenging activity of SIRPI in physiological condition make SIRPI eliminate partially generated ^1^O_2_. This double‐edged performance results in the relatively low ^1^O_2_ generation in physiological environment and limited ^1^O_2_ generation in normal tissue, diminishing the potential side effects during treatment.

### 2.4 ROS Generation of SIRPI in Mimic Tumor Microenvironment (TME)

**Figure 3:**
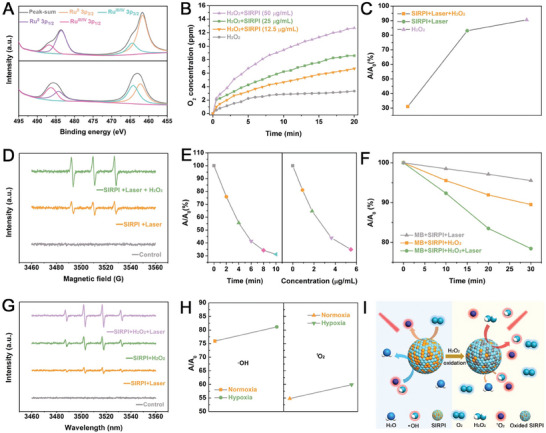
Multiple ROS generation of SIRPI in mimic tumor environment (TME). A) XPS high‐resolution spectra of Ru 3p in SIRPI without and with H_2_O_2_ incubation (Top: without H_2_O_2_; bottom: with H_2_O_2_). B) CAT‐like activity of SIRPI to accelerate the decomposition of H_2_O_2_ to generate O_2_. C) The absorbance of DPBF at 665 nm and D) ESR spectra of TEMP/ ^1^O_2_ to analyze the ^1^O_2_ generation efficiency by SIRPI under different treatments. E)The absorbance of DPBF at 665 nm with different times of irradiation and different concentration of SIRPI to explore the ^1^O_2_ generation rate. F) The absorbance of MB at 665 nm and G) ESR spectra of DMPO/•OH to analyze the •OH generation efficiency by SIRPI under different treatments. H) The ability of SIRPI to generate •OH and ^1^O_2_ under normoxia and hypoxia conditions, respectively, which is used to assess the CAT‐like activity of SIRPI overcome the limitation for SIRPI on ROS generation. I) Schematic illustration of the switchable ROS generation and scavenging in response with H_2_O_2_.

TME is characterized by mild acidity, high H_2_O_2_ concentration, and massive enrichment of GSH, distinguishing it from the normal tissues.^[^ ^17^ ^]^ To explore the specific responsiveness of SIRPI to the TME, we investigated the structural changes of SIRPI in mimic TME. The XPS analyses show a notable increase in the valence states of Ru element in SIRPI in the mimic TME with high H_2_O_2_ level compared to the physiological environment. The main valence state forms of Ru changed from reductive Ru^0^ to oxidative Ru^3+/4+^ (**Figure** **3**A). This H_2_O_2_ responsive structural and valence change of SIRPI is promising to specifically generate ROS based the oxidation state in tumor.^[^ ^18^ ^]^ Meanwhile, we noted that SIRPI exhibited strong catalase‐like (CAT‐like) activity to accelerate the decomposition of H_2_O_2_ to generate O_2_ within a very short time (Figure 3B). This efficient CAT‐like activity is highly promising to alleviate the hypoxic TME and provides sufficient O_2_ to increase the ^1^O_2_ generation in tumor for augmenting the efficiency of tumor therapy.^[^ ^19^ ^]^ With the above hypothesis we further investigated the ^1^O_2_ generation of SIRPI in the presence of H_2_O_2_. Interestingly, we observed significantly absorbance decrease of DPBF at 412 nm after introducing H_2_O_2_ (Figure 3C; Figures S4–S6, Supporting Information). This phenomenon could be attributed to the synergy between the elevated valence of Ru and enhanced O_2_ supplying based on CAT‐like activity_,_ which result in the decrease of ROS elimination and provide sufficient O_2_ as the free electron receptor to improve the ^1^O_2_ generation efficiency. Consistent with the UV–vis analyses, the signal intensity of TEMP/^1^O_2_ in ESR spectra increased from the group without H_2_O_2_ to the group with H_2_O_2_, proving the efficient H_2_O_2_ responsive ^1^O_2_ generation of SIRPI (Figure 3D). Moreover, the ^1^O_2_ generation of SIRPI shows a significant time and concentration dependence in the presence of H_2_O_2_ under NIR laser irradiation. SIRPI enables to generate abundant ^1^O_2_ in a short time (less than 10 min) with low concentration (7–11 µg mL^−1^), confirming that SIRPI endorses an efficient ^1^O_2_ generation ability in the mimic TME with high H_2_O_2_ level (Figure 3E).

Since Fe^2+^ and Ru^3+^ have been proved to undergo Fenton and Fenton‐like reactions to generate •OH in tumor environments, we employed methylene blue (MB), a •OH probe, to test the •OH generation by SIRPI. The UV‐vis spectra show a significant decrease in the absorbance of MB at 665 nm after incubation with SIRPI and H_2_O_2_ for 30 minutes, suggesting that SIRPI performs the excellent •OH generation by Fe^2+^ and Ru^3+^. Notably, the •OH generation rate is further elevated by NIR laser irradiation (Figure 3F; Figure S7, Supporting Information). This phenomenon suggests that NIR laser irradiation would not merely as the stimulator to generate ^1^O_2_, but also accelerates the ROS production rate by increasing the temperature based on the mild photothermal conversion ability of SIRPI (Figure S8, Supporting Information). Additionally, the ESR spectra show obvious DMPO/•OH signals in the group treated by SIRPI and H_2_O_2_, especially with NIR laser irradiation, confirming the excellent performance of SIRPI to generate •OH synergistically (Figure 3G). These results strongly suggest that SIRPI is expected to be diverse and large amounts of ROS producer in mimic TME. Moreover, the ^1^O_2_ and •OH generation efficiency of SIRPI is not dramatically weakened by exposing to the hypoxic condition, which is derived from the cooperative effect of CAT‐like activity and indicate the potential of SIRPI to overcome hypoxia environment to achieve high tumor therapeutic efficacy (Figure 3H; Figures S9 and S10, Supporting Information). Therefore, SIRPI is prospective for stable, specific, and highly efficient ROS generation in TME (Figure 3I).

### 2.5 Differentially Control the ROS Levels and Specific Pyroptosis by SIRPI In Vitro

**Figure 4:**
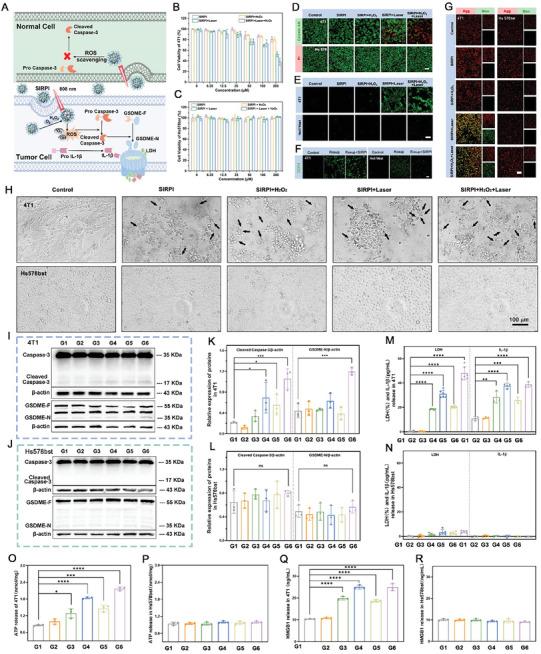
The anti‐tumor effect and ROS generation‐guided specific pyroptosis by SIRPI in vitro. A) Schematic illustration of the multiple ROS generation guided specific pyroptosis in normal and tumor cells by SIRPI. The relative viability of B) 4T1 cells and C) Hs578bst cells with different treatments (*n* = 4). D) The Calcein‐AM (live cells) and PI (dead cells) staining after different treatments, the scale bar is 100 µm. E) Fluorescence images of DCFH‐DA staining in 4T1 cells and Hs578bst cells treated differently to detect ROS generation, the scale bar is 100 µm. F) Fluorescence images of 4T1 cells and Hs578bst cells incubated with Rosup, and Rosup + SIRPI using DCFH‐DA as indicator, the scale bar is 100 µm. G) Fluorescence images to detect mitochondria membrane potential measurement by JC‐1 probe, the scale bar is 100 µm. H) The morphology observation of 4T1 cells and Hs578bst cells with different treatment, the scale bar is 100 µm. Western blot analysis of the expressions of cleaved caspase‐3 and GSDME‐N in I) 4T1 cells and J) Hs578bst cells under different treatment regimens. Quantitative analysis of cleaved caspase‐3 and GSDME‐N expression in K) 4T1 cells and L) Hs578bst cells based on the western blot results (*n* = 3). The release of LDH and IL‐1β from M) 4T1 cells and N) Hs578bst cells after different treatments (*n* = 5). ATP release from O) 4T1 cells and P) Hs578bst cells after different treatments (*n* = 3). HMGB1 release from Q) 4T1 cells and R) Hs578bst cells after different treatments (*n* = 3). From G1 to G6, they are Control, Laser, SIRPI, SIRPI + Laser, SIRPI + H_2_O_2_, SIRPI + Laser + H_2_O_2_, respectively. Data are present as mean ± SD, *p < 0.05, **p < 0.01, and ***p < 0.001, ****p < 0.0001, compared to the Control group.

ROS accumulation is enables to induce pyroptosis via the caspase‐3/gasdermin E (GSDME) pathway, resulting in cell membrane swelling and the releases of damage‐associated molecular patterns (DAMPs) and inflammatory cytokines.^[^ ^4^, ^10^ ^]^ Given the specific ROS generation feature of SIRPI in the mimic TME, a systematic mechanism of specific pyroptosis in normal and tumor cells by SIRPI is proposed (**Figure** **4**A). Initially, we studied the intracellular uptake behavior of SIRPI. To visualize the intracellular uptake, we prepared SIRPI labeled with Rhodamine B. As shown in Figure S11 (Supporting Information), a strong red fluorescent signal is observed after treatment in 4T1, MCF‐7, and Hs578bst cells treated by Rhodamine B‐labeled SIRPI, indicating that SIRPI is successfully internalized by the cells. As the first organelle after the SIRPI entry, it is crucial to understand whether SIRPI escapes from the lysosome.^[^ ^20^ ^]^ To verify the lysosomal escape ability of SIRPI, we performed confocal imaging using Rhodamine B‐labeled SIRPI and the lysosomal marker (Lyso‐Tracker). Fluorescence images show that the red fluorescent signal (Rhodamine‐labeled SIRPI) overlap heavily with the green fluorescent signal (Lyso‐Tracker) at 2 h of SIRPI incubation, suggesting the successful internalization of SIRPI by the lysosomes. Additionally, as the incubation time of SIRPI increased to 6 h, the co‐localization of the red fluorescence with the green fluorescence decreased, indicating the SIRPI successfully escapes from the lysosomal, which is expected to be effective in inhibiting tumor cells growth (Figure S12, Supporting Information). Based on these findings, we assessed the cytotoxicity of SIRPI on tumor cells (4T1 and MCF‐7) and normal cells (Hs578bst) under different conditions by cell‐counting‐kit‐8 (CCK‐8) assay. The viability of tumor cells gradually decreased with the increase of SIRPI concentration in all groups, especially in the SIRPI + Laser + H_2_O_2_ group (Figure 4B; Figure S13, Supporting Information). Notably, the viability of 4T1 cells reduced to 40% with the treatment of H_2_O_2_ and 200 µM SIRPI under NIR laser irradiation, indicating the potent anti‐tumor efficacy of SIRPI. Conversely, there is negligible inhibition observed in Hs578bst cells with the same treatment, even in the group treated with both H_2_O_2_ and Laser (Figure 4C). These exciting results reveal the minimal side effect of SIRPI in normal cells and imply its high biocompatibility in vitro. Consistent with the CCK‐8 analyses, the fluorescence images staining by Calcein acetoxymethyl ester (Calcein‐AM) and propidium iodide (PI) display a strong red and weak green fluorescence in 4T1 cells treated by SIRPI and H_2_O_2_ under irradiation. In contrast, the Hs578bst cells treated with the same strategy exhibit a strong green fluorescence (Figure 4D). These findings indicate that SIRPI displays negligible cytotoxicity to normal cell under the same treatment conditions, suggesting its promising potential safety in normal cells while showing potential act as an effective anti‐tumor agent in tumor cells.

To verify the specific anti‐tumor effect of SIRPI resulting from the differentially control of ROS levels in tumor and normal cells, we monitored the ROS levels in both 4T1 and Hs578bst cells using the 2,7‐dichlorofluorescein diacetate (DCFH‐DA) as probe. The fluorescence images of 4T1 cells treated by SIRPI show visible green fluorescence. The most significant green fluorescence is detected in the SIRPI + Laser + H_2_O_2_ group. Conversely, there is no apparent green fluorescence could be seen in the Hs578bst cells with the same treatment (Figure 4E). These results indicate that the ROS generation of SIRPI is specifically dependent on TME. To demonstrate the simultaneous generation of •OH and ^1^O_2_ by SIRPI in 4T1 cells, we chose 2',7'‐dichloro‐3'‐hydroxy‐6'‐(4‐hydroxy‐3,5‐diiodophenoxy) spiro [2‐benzofuran‐3,9'‐xanthene]‐1‐one (HKOH‐1r) and singlet oxygen sensor green (SOSG) as probe to detect the intracellular •OH and ^1^O_2_ accordingly. Consistent with the DCFH‐DA staining, we observed a remarkable green fluorescence in tumor cells treated with SIRPI by both HKOH‐1r and SOSG staining, indicating the diverse ROS generated by SIRPI in tumor cells (Figure S14, Supporting Information). To confirm the precisely control of ROS levels in tumor and normal cells of SIRPI, we studied the ROS elimination efficiency in 4T1 and Hs578bst cells using Rosup to increase the ROS levels. Notably, the fluorescent intensity generated by the Rosup stimulation remarkably decreased in the presence of SIRPI in Hs578bst cells, directly showing the ROS scavenging activity of SIRPI in normal cells. Conversely, we noticed a slight increase on fluorescent intensity in 4T1 cells treated with SIRPI, indicating that SIRPI enables a sensitive response to the TME with structural alterations, presenting efficient ROS‐generating activity (Figure 4F). Subsequently, we evaluated the H_2_O_2_ content in both Hs578bst cells and 4T1 cells. As expected, the H_2_O_2_ content in 4T1 cells is about 2.9 times higher than that in Hs578bst cells (Figure S15, Supporting Information). This obvious different H_2_O_2_ levels in normal and tumor cells enables SIRPI to work as a ROS scavenger in normal cells for ROS elimination to minimize the side effect, while acting as a ROS generator in tumor cells for varying ROS generation to efficiently damage tumor cells. Mitochondria, as the energy supplier in cells, is sensitive to ROS offense and presents a decreased membrane potential in response to ROS damage.^[^ ^21^ ^]^ We monitored the mitochondrial membrane potential changes in different groups using 5,5,6,6‐tetrachloro‐1,1,3,3‐tetraethyl‐imidacarbocyanine iodide (JC‐1) as probe. The fluorescence images show significantly decrease in red fluorescent aggregates while increase in green fluorescent monomer in 4T1 cells treated with SIRPI + Laser and SIRPI + Laser + H_2_O_2_. Whereas no significant changes are observed in Hs578bst cells with similar treatments (Figure 4G). These results suggest that SIRPI induces a large amount of ROS and effectively reduces the mitochondria membrane potential in tumor cells, while protecting the mitochondria from ROS damage in normal cells.

Since the SIRPI differentially control the ROS levels in tumor or normal cells, we explore its capacity to induced specific pyroptosis in tumor cells. Notably, a typical membrane swelling is observed in 4T1 cells, which tend to be more marked following stimulation with H_2_O_2_ and Laser irradiation. In contrast, the Hs578bst cells maintained their typical morphology without exhibiting any pyroptosis features with the same treatment (Figure 4H). To systematically evaluate the specific pyroptosis, we assessed the expression levels of cleaved caspase‐3 and GSDME‐N in tumor cells (4T1 and MCF‐7) and normal cells (Hs578bst) cells with different treatment by western blot assay. It appears that cleaved caspase‐3 and GSDME‐N expression levels are significantly upregulated in the 4T1 and MCF‐7 cells treated by SIRPI, especially in the group of SIRPI and H_2_O_2_ under NIR laser irradiation (Figure 4I, K; Figures S16‐S18, Supporting Information). Conversely, no apparent changes are detected in Hs578bst cells treated by SIRPI (Figure 4J, L; Figure S19, Supporting Information). Since lactate dehydrogenase (LDH) and IL‐1β are typical markers of pyroptosis, we detected the release of LDH and IL‐1β from 4T1 and Hs578bst cells after different treatments. Remarkably, 4T1 cells treated by SIRPI shows significant increase of LDH and IL‐1β release. The release ratio in the group of SIRPI + Laser + H_2_O_2_ is as high as 48.1% for LDH and 38.6 (pg mL^−1^) for IL‐1β, which is significantly higher than control group and suggest the notable pyroptosis in 4T1 cells treated by SIRPI (Figure 4M). Whereas the released LDH and IL‐1β in Hs578bst cells with same treatments are below 5% (Figure 4N). These results clearly demonstrate that SIRPI successfully induce pyroptosis in tumor cells, while maintain pyroptosis silence in normal cells. Additionally, pyroptosis enabled the release of DAMPs, including adenosine triphosphate (ATP) and high mobility group protein B1 (HMGB1), which exposing the massive antigen epitope and enhancing the immunogenicity.^[^ ^22^ ^]^ We examined the release of ATP and HMGB1 in 4T1 and Hs578bst cells with different treatments as well. SIRPI results in an obvious increase in the release of ATP and HMGB1 in 4T1 cells, especially the group SIRPI + Laser + H_2_O_2_ (Figure 4O, Q). Nevertheless, the released of ATP and HMGB1 in Hs578bst cells treated by SIRPI remained within normal ranges (Figure 4P, R). There results directly indicate that SIRPI enables specifically enhance the immunogenicity of tumor cells and is expected to augment the anti‐tumor immune response. Besides, the cell death is typically triggered by multiple mechanisms acting synergistically. Therefore, we explored its potential to induce cell death through the apoptotic pathway in addition to its ability to selectively induce tumor cell pyroptosis. Flow cytometry analyses reveal that SIRPI significantly activate the apoptotic pathway in 4T1 cells (Figure S20, Supporting Information). These findings suggest that SIRPI acts through multiple mechanisms to inhibit tumor cells, offering stronger support for its role in precise tumor therapy.

### 2.6 Pyroptosis Induced by SIRPI in Hypoxia Condition

**Figure 5:**
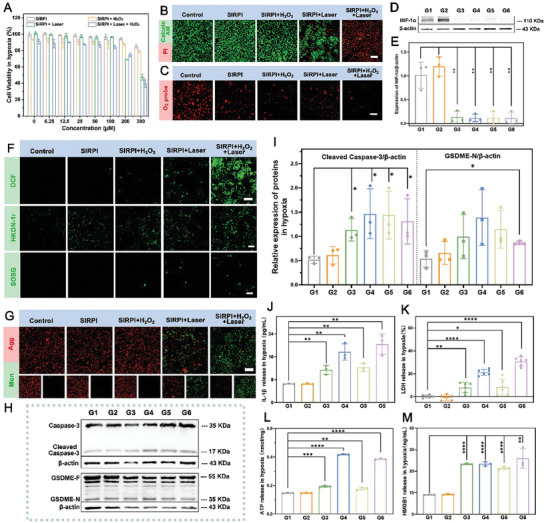
TME amelioration of SIRPI in vitro. A) Relative cell viability of 4T1 cells with different treatments incubated with 1% O_2_ (*n* = 4). Fluorescence images of B) Calcein‐AM/PI staining and C) [Ru(dpp)_3_]^2+^Cl staining of 4T1 cells with different treatments under hypoxia condition, the scale bar is 100 µm. D) Western blot result to analysis the expression levels of HIF‐1α under different treatment for hypoxia amelioration detection. E) Quantitative analysis of HIF‐1α expression in 4T1 cells incubated with 1% O_2_ based on the western blot results (*n* = 3). Fluorescence images of F) DCFH‐Da, HKOH‐1r, and SOSG staining (to detect the generations of ROS, •OH, and ^1^O_2_, respectively), G) JC‐1 staining in hypoxia incubation, the scale bar is 100 µm. H) Expressions of cleaved caspase‐3 and GSDME‐N in 4T1 cells under hypoxia condition using western blot analysis. I) Quantitative analysis of cleaved caspase‐3 and GSDME‐N expression of 4T1 cells in hypoxia condition based on the western blot results (*n* = 3). The release of J) IL‐1β, K) LDH, L) ATP, and M) HMGB1 from 4T1 cells after different treatments in 1% O_2_ condition. (*n* = 3). From G1 to G6, they are Control, Laser, SIRPI, SIRPI + Laser, SIRPI + H_2_O_2_, SIRPI + Laser + H_2_O_2_, respectively. Data are present as mean ± SD, *p < 0.05, **p < 0.01, and ***p < 0.001, ****p < 0.0001, compared to the Control group.

Hypoxia, a specificity of the tumor microenvironment, significantly limit the ROS induced tumor pyroptotic death. Based on the CAT‐like activity of SIRPI, we assessed the hypoxia ameliorate ability and pyroptotic tumor cell death induced by SIRPI under hypoxic conditions. The CCK‐8 assay indicates that the viability of 4T1 cells is remarkably inhibited by SIRPI, especially the group treated with both H_2_O_2_ and Laser (**Figure** **5**A). The fluorescence images of Calcein‐AM and PI staining images show that the red fluorescence almost completely replaced the green fluorescence in the group of SIRPI + Laser + H_2_O_2_ (Figure 5B). These results suggest that SIRPI with CAT‐like activity catalyzes H_2_O_2_ into O_2_, providing sufficient O_2_ to alleviate hypoxic and generate ^1^O_2_ to synergistically inhibit tumor cells progression. To verify this hypothesis, we investigated the tumor hypoxia regulation of SIRPI. The SIRPI treatment cause a decreased red fluorescence in the O_2_ level assessment using [Ru(dpp)_3_] Cl_2_ as indicator, showing the increase of O_2_ concentration in 4T1 cells (Figure 5C). Besides, the 4T1 cells treated by SIRPI show downregulated expression of HIF‐1α compared to the 4T1 cells treated by phosphate buffer solution (PBS) or NIR laser (Figure 5D, E; Figure S21, Supporting Information). These results verify the effectiveness of SIRPI to increase the O_2_ level and regulate the hypoxia of tumor. Subsequently, we tested the ROS generation in 4T1 cells by SIRPI. It appears that significant green fluorescence could be observed in the cells treated by SIRPI in DCFH‐DA, HKOH‐1r, and SOSG staining, revealing the effective •OH and ^1^O_2_ generation (Figure 5F). Meanwhile, the fluorescence images of JC‐1 staining exhibit a remarkable green fluorescence in the cells treated with SIRPI, demonstrating the generation of ROS effectively reduced the mitochondrial membrane potential of tumor cells in the hypoxia condition (Figure 5G). To investigate the pyroptosis induced by SIRPI under hypoxia, we assessed the expressions of cleaved caspase‐3 and GSDME‐N in 4T1 cells using western blot. The expression levels of cleaved caspase‐3 and GSDME‐N in cells treated by SIRPI are significantly higher than the cells treated by PBS and laser (Figure 5H, I; Figure S22, Supporting Information). Besides, the cells treated by SIRPI show typical membrane swelling and increased LDH and IL‐1β release compared to the cells treated by PBS and NIR laser irradiation. These results clearly show the noticeable pyroptosis induced by SIRPI under hypoxia, particularly following stimulation with H_2_O_2_ and laser (Figure 5J, K; Figure S23, Supporting Information). Meanwhile, the cells treated with SIRPI in hypoxia condition exhibit substantial ATP and HMGB1 release. These findings imply the effective performance of SIRPI to ameliorated tumor hypoxic environment and induce tumor pyroptosis to release DAMPs, enhancing the immunogenicity of tumor cells and potentially inhibiting the development of metastases (Figure 5L, M).

### 2.7 RNA Sequence Analyses of SIRPI

**Figure 6:**
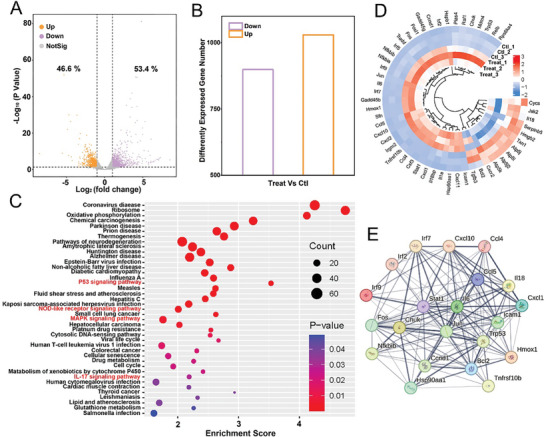
RNA Sequence Analysis of SIRPI. A) Volcano plots and B) numbers of the differentially expressed genes between Control and SIRPI + Laser + H_2_O_2_ group. C) Diagram of KEGG pathway enrichment analysis. D) Circus heatmap of related DEGs associate with oxidative stress and inflammation E) Protein‐protein interaction network. Treat is the SIRPI + Laser + H_2_O_2_‐treated group and Ctl is the PBS‐treated group.

To investigate the mechanism of SIRPI triggered pyroptosis, we performed transcriptome sequencing analyses of 4T1 cells from Control and SIRPI + Laser + H_2_O_2_ groups. In comparison with the Control group, there are 1030 (53.4%) upregulated genes and 899 (46.6%) downregulated genes in the SIRPI + Laser + H_2_O_2_ group (**Figure** **6**A, B; Figure S24A, Supporting Information). Subsequent Kyoto Encyclopedia of Genes and Genomes (KEGG) analyses show that the differentially expressed genes (DEGs) associate with SIRPI are mainly enriched in inflammatory signaling pathways, such as the oxidative phosphorylation, p53 signaling pathway, NOD‐like receptor signaling pathway, MAPK signaling pathway, and IL‐17 signaling pathway (Figure 6C). These results suggest the close association among the tumor cells inhibitory effect of SIRPI and the inflammatory response induced by oxidative stress.^[^ ^4a^ ^]^ Subsequently, we selected the top 20 DEGs among the above pathways to perform gene ontology (GO) enrichment analyses. As expected, the results confirm the association between the DEGs and the oxidative stress and inflammatory pathways, verifying the critical effect of SIRPI as ROS‐generator in tumor suppression (Figure S24B, Supporting Information). For systematic investigation of 4T1 cells inhibition by SIRPI triggered pyroptosis and inflammatory response, we extracted a subset of the DEGs to perform circus heatmap. Notably, the levels of Irf7, Hmox1, Trp53, Fos Nfkbib, Cxcl1, Il6, Ccl5, etc., are markedly upregulated, indicating significant enhancement of oxidative stress and inflammation caused by SIRPI. Additionally, the downregulation of Atp5j, Atp5k, Atp5l, Jun, etc., suggest that mitochondrial oxidative stress induced by SIRPI effectively reduces the mitochondrial membrane potential and inhibit tumor cell growth (Figure 6D). Based on the protein‐protein interaction (PPI) network analyses on relevant DEGs, we identified the correlation between oxidative stress, inflammatory response, and tumor suppression induced by SIRPI (Figure 6E). However, the transcriptomic sequencing only provides valuable insights into gene expression, which is not fully reflective of protein levels and other downstream effects. Therefore, we confirmed the ability of SIRPI as a ROS‐generator to specifically increase oxidative stress in tumor cells, boosting the inflammatory response in the tumor region, and effectively inducing specific pyroptotic tumor cell death by combining the transcriptome sequencing with western blot analysis of pyroptosis‐related proteins.^[^ ^23^ ^]^

### 2.8 Specific Pyroptosis by SIRPI In Vivo

**Figure 7:**
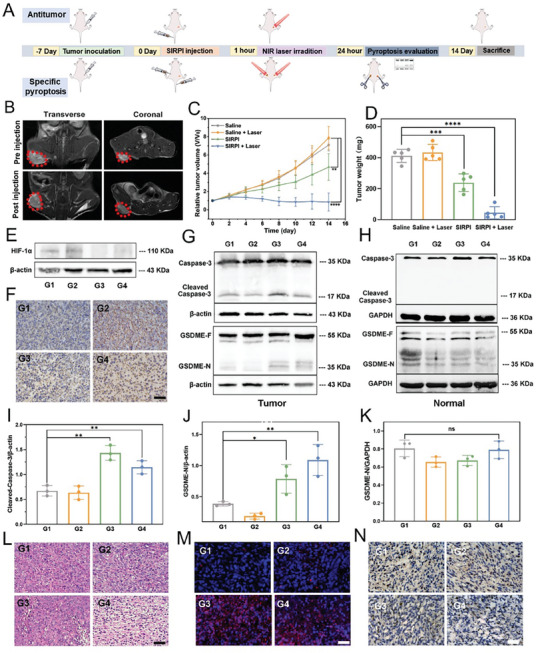
Antitumor effect and specific pyroptosis of SIRPI in vivo. A) Experimental scheme of SIRPI for the antitumor effect and specific pyroptosis. B) *T*_2_‐weighted MR images of tumor bearing mice before after 1 h intravenous injection of SIRPI. C) Relative tumor volume, D) tumor weight of 4T1 tumor‐bearing mice with different treatments. E) Western blot analysis and F) immunohistochemistry images to evaluate the expression of HIF‐1α in the tumor tissues after differently treated, the scale bar is 50 µm. Protein expressions of cleaved caspase‐3 and GSDME‐N with different treatment in G) tumor and H) normal tissue. Quantitative analysis of I) cleaved caspase‐3 and J) GSDME‐N expression in tumors (*n* = 3). K) Quantitative analysis of GSDME‐N expression in normal tissue (*n* = 3). L) H&E, M) TUNEL, and N) Ki67 staining of tumors section from each group, the scale bar is 50 µm. From G1 to G4, they are Saline, Saline + Laser, SIRPI, SIRPI + Laser, respectively. Data are present as mean ± SD, *p < 0.05, **p < 0.01, and ***p < 0.001, ****p < 0.0001, compared to the Saline group.

Before investigating the antitumor effect of SIRPI, we first analyzed biosafety of SIRPI in vivo. The hemolysis analyses show that the hemolysis rate of erythrocytes incubated with SIRPI remained within the safe range (less than 5.0%) even at a concentration of 200 µM (Figure S25, Supporting Information). Consequently, intravenous administration of SIRPI in mice were followed by monitoring the biochemical indexes after 24 h. It appears that the main biochemical indexes, including aminotransferase (AST), alanine aminotransferase (ALT), alkaline phosphatase (ALP), blood urea nitrogen (BUN), creatinine (CR), and uric acid (UA), in the SIRPI and Saline treated mice are within the normal range (Figure S26, Supporting Information). Moreover, the hematoxylin‐eosin (H&E) staining of the major organs (heart, liver, spleen, lung, and kidney) in mice treated by SIRPI and laser display no significant histological damage, suggesting the superior biosafety and biocompatibility of SIRPI (Figure S27, Supporting Information). To further verify the accumulation of SIRPI in tumor through enhanced permeability and retention effect, we quantified the uptake of SIRPI in tumors by inductively coupled plasma‐mass spectrometry (ICP‐MS) and Prussian blue staining. It appears that sufficient SIRPI is detected in tumor of mice treated by SIRPI, revealing the enrichment of SIRPI in tumor regions (Figures S28 and S29, Supporting Information). Based on the excellent *T*_2_ contrast imaging performance of SIRPI in vitro, we proceeded to assess its imaging ability in solid tumors (Figure S30, Supporting Information). *T*_2_‐weighted MR images acquired before and after injection of SIRPI reveal a noticeably darkened signal in the tumor region post‐injection (**Figure** **7**B). The signal‐to‐noise ratio (SNR) values following injection are about 69.8 and 80.9% in transverse and coronal planes (Figure S31, Supporting Information). This significant contrast between tumor and normal tissue endows us to easily differentiate the tumor from surrounding normal tissue, which is promising for real‐time monitoring of the tumor therapy.

Subsequently, we assessed the tumor therapeutic efficacy of SIRPI in vivo (Figure 7A). The female 4T1 tumor‐bearing Balb/c mice were randomly assigned into 4 groups (*n* = 5): Saline, Saline + Laser, SIRPI, and SIRPI + Laser group. During the treatment, the mice weight fluctuates within the normal range with negligible difference among all groups (Figure S32A, Supporting Information). We noticed that the tumor growth is significantly inhibited in SPIRI treated group, especially the mice subjected to SPIRI and NIR laser combination therapy (Figure 7C, D; Figure S32B–D, Supporting Information). To evaluate the aggressive effect of SIRPI on ameliorate the tumor hypoxia environment, we evaluated the hypoxia levels in tumors of mice with different treatments by western blot and immunohistochemistry staining for HIF‐1α. It appears that the HIF‐1α expression in the SIRPI‐treated group is obviously lower than that in the Saline group, indicating the excellent CAT‐like activity of SIRPI in tumor tissues to alleviate the hypoxic condition and enhancing the therapeutic efficiency (Figure 7E, F; Figures S33 and S34, Supporting Information). Additionally, we analyzed the ROS levels in both tumor and normal tissues under different treatments. Dihydroethidium (DHE) staining results show a significantly increased ROS level in tumor tissues treated with SIRPI + Laser, while no significant changes were observed in normal tissues. The substantial ROS generation in tumor tissues is expected to mediate selective pyroptosis in tumor cells (Figure S35, Supporting Information). To verify the significant pyroptosis in tumor induced by SIRPI in vivo, we detected the expression of cleaved caspase‐3 and GSDME‐N in tumor tissues by western blot analyses following intravenous administration of SIRPI. The expression of cleaved caspase‐3 and GSDME‐N in tumor tissue are significantly upregulated in response to the SIRPI as compared to the Saline group. In particularly, there is an elevation of nearly 1.7 and 3.8 times of cleaved caspase‐3 and GSDME‐N (Figure 7G, I, J; Figure S36, Supporting Information). Meanwhile, we also analyzed the expression of cleaved caspase‐3 and GSDME‐N in normal tissue. Interestingly, we do not notice apparent change of cleaved caspase‐3 and GSDME‐N levels in mice treated with SIRPI compared to the mice treated with saline, indicating that SIRPI specifically induces the upregulation of pyroptosis associated proteins in tumor tissues while maintaining normal levels in normal tissues (Figures S37–S39, Supporting Information). These results demonstrate that SIRPI enables to precisely inhibit the growth of tumor through the pyroptosis pathway in vivo without pyroptosis‐inducing effect in normal tissues. However, the precise pyroptosis in tumor may be caused by the different accumulation amount of SIRPI in tumor and normal tissue. To investigate whether SIRPI induces pyroptosis in tumor tissues and maintaining pyroptosis silence in normal tissues by the switchable activity in response with different tissues, we subcutaneously injected an equal amount of SIRPI into normal tissues with laser irradiation and analyses the expression levels of cleaved caspase‐3 and GSDME‐N domain. The western blot analyses indicate that the SIRPI result in the negligible expression of cleaved caspase‐3 and GSDME‐N increase compared to the Saline group, even in the SIRPI + Laser group (Figure 7H, K; Figure S40, Supporting Information). These results strongly prove that SIRPI shows pyroptosis silencing in normal tissues, demonstrating SIRPI enables the excellent property to induce selective cellular pyrolysis in the tumor region while negligible damage to the normal tissue. Additionally, we performed the H&E, TUNEL, and Ki67 staining to systematically evaluate the therapeutic effects of SIRPI. The H&E and TUNEL staining images show that SIRPI + Laser treatment severely damage tumor tissue structure (Figure 7L, M). The Ki67 expression in the SIRPI + Laser group is significantly lower than that in the Saline group, indicating the tumor cells proliferation is inhibited (Figure 7N). These results synergistically demonstrate the effective anti‐tumor effect of SIRPI.

### 2.9 Augmented Immune Effects Mediated by SIRPI in Inhibiting Lung Metastasis

**Figure 8:**
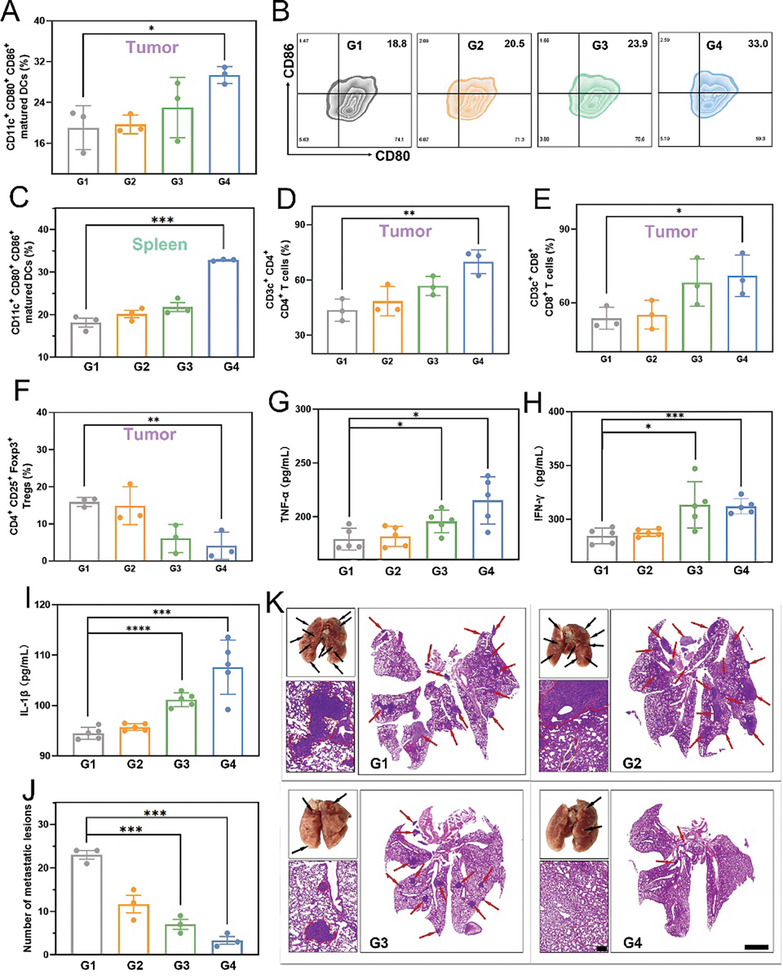
Augmented immune effects by SIRPI. A) Quantification of the proportions of CD80^+^ CD86^+^ DCs in the tumor (*n* = 3). B) Flow cytometry analyses of CD80^+^ and CD86^+^ on CD11c^+^ cells in spleen after different treatments. C) Quantification of the proportions of CD80^+^ CD86^+^ DCs in the spleen (*n* = 3). D) Quantification of the proportions of CD4^+^ T cells, E) CD8^+^ T cells, and F) CD25^+^ Foxp3^+^ Tregs in the tumor (*n* = 3). ELISA analysis of G) TNF‐α, H) IFN‐γ, and I) IL‐1β concentration in tumor‐bearing Balb/c mice serum after differently treated for 24 h (*n* = 5). J) Statistics on the metastases number in lung at 30 days after different treatments (*n* = 3). K) H&E staining images of metastatic lesions of different treatment group. The metastatic regions were marked by the black arrows, red circles, and red arrows, the scale bar is 200 µm and 2 mm, respectively. From G1 to G4, they are Saline, Saline + Laser, SIRPI, SIRPI + Laser, respectively. Data are present as the mean ± SD, *p < 0.05, and ***p < 0.001, ****p < 0.0001, compared to the Saline group.

Building on the successful anti‐tumor effects of SIRPI, we explored the potential of SIRPI to enhance immune responses within the TME. Initially, we analyze the dendritic cells (DCs) maturation by assessing the expression of DC‐related markers CD80 and CD86 in both the spleen and tumor tissues. Flow cytometry results display that expression of CD80^+^ and CD86^+^ in the SIRPI‐treated group is significantly higher than that treated by Saline, implying an increase in DCs maturation (**Figure** **8**A–C; Figure S41A, Supporting Information). Specifically, the DCs maturation ratio in SIRPI + Laser group is about 33.0% compared to the 18.8% in the Saline group in spleen, suggesting that the SIRPI + Laser synergistic therapy effectively promotes the maturation of DCs. Next, to systematically detect the immune cell infiltration, we conducted flow cytometry to quantify the various immune cells infiltration in the tumors.^[^ ^9^, ^24^ ^]^ These results reveal a significant increase in the proportion of CD4^+^ and CD8^+^ T cells in the SIRPI + Laser group, whereas the proportion of immunosuppressive regulatory T cells (Tregs) characterized by CD25 and Foxp3 expression was significantly reduced (Figure 8D–F; Figure S41B–D, Supporting Information). Moreover, the immunofluorescence staining images of CD4^+^ and CD8^+^ T cells infiltration in the tumor tissues corroborated this finding, shows a significant increase in the intensity of red (CD4^+^) and green (CD8^+^) fluorescence in the SIRPI‐treated group compared to the Saline group (Figure S42, Supporting Information). These findings demonstrate that SIRPI‐induced pyroptosis significantly promotes DCs maturation and reversing the immunosuppressive in TEM. However, while increased infiltration of CD4+ and CD8+ T cells is evident, we acknowledge that their specific activation or function in the tumor region remains to be further explored. Additionally, the levels of TNF‐α, IFN‐γ, and IL‐1β in the SIRPI‐treated group, especially in the synergistic treated by SIRPI and laser irradiation, are significantly higher than those in the Saline group (Figure 8G–I). These results confirm that SIRPI successfully promotes the expression of inflammatory factors and improving the systemic immune response, which is expected to inhibit the development of micro‐metastases. To further investigate the potential of SIRPI in inhibiting metastasis, we injected 4T1 cells into mice with multiple interventions of SIRPI to establish a metastatic tumor model. After 2 weeks, a large number of micro‐metastatic foci are visible in the lung tissues of the Saline‐treated group. The number of micro‐metastatic foci in Saline‐treated group is significantly higher than that in SIRPI‐treated group. Notably, it hard to observe typical metastases in SIRPI + Laser group, indicating the high efficiency of SIPRI on reduce the risk of metastases during tumor therapy (Figure 8J; Figure S43, Supporting Information). Consistent with the optical images analyses, the H&E staining images of the lungs and the calculated ratio of metastatic tumors areas in the total lung tissue area indicate that the SIRPI treatment could effectively inhibit the metastases (Figure 8K; Figure S44, Supporting Information). These findings confirm that SIRPI enhances cellular immunogenicity and increases the inflammatory factor levels through the specific pyroptosis, re‐modeling the immunosuppressive microenvironment by promoting DCs maturation and tumor T cell infiltration, thereby effectively inhibiting primary tumor growth and metastatic nodes.

## 3 Conclusion

In this work, we have developed a switchable reactive oxygen species (ROS) generator/ scavenger in response with hydrogen peroxide (H_2_O_2_) to trigger the specific pyroptosis in tumors, effectively promoting therapeutic efficiency. This intelligent inducer performs reductive properties to eliminate excessive ROS and minimize the side effects with the excellent biosafety in normal tissues. Notably, in tumor microenvironment (TME) with H_2_O_2_ accumulation, the structure of specific iron ruthenium pyroptosis inducer (SIRPI) is altered and switched to an ROS generator, which specifically generate large amounts of singlet oxygen (^1^O_2_) and hydroxyl radical (**·**OH) and trigger the specific pyroptotic tumor cell death via the caspase‐3/ gasdermin E (GSDME) pathway. This mode of programmed cell death effectively boosts inflammatory factors and damage‐associated molecular patterns (DAMPs) release, alleviates the immunosuppressive TME, enhances the immune response, and suppresses primary and metastatic tumors. Therefore, the selective pyroptosis in tumor cells mediated by structural alterations in the specific response of SIRPI to H_2_O_2_ is anticipated to offer a novel strategy for the precisive tumor therapy.

## 4 Experimental Section

Experimental details are provided in the Supporting Information.

### Statistical Analysis

Statistical analysis was attained by Graphpad Prism 8.0 and the statistical mode was analysis of one‐/two‐way analysis of variance (ANOVA) and Student's t‐test, the data are presented as mean + SD. *p* values of less than 0.05 was considered significant (*p < 0.05, **p < 0.01, ***p <0.001, and ****p < 0.0001).

## Conflict of Interest

The authors declare no conflict of interest.

## Author Contributions

L.H., C.T., and Z.Z. conceived and designed the experiments. Z.Z., L.H., S.Z., M.L., M.T., M.F., J.Z., J.Z., M.L., K.L., and C.T. performed the experiments. L.H., K.L., C.T., and Z.Z. analyzed the data, and L.H. and Z.Z. co‐wrote the paper. All authors discussed the results and commented on the manuscript.

## Supporting information



Supporting Information

## Data Availability

The data that support the findings of this study are available from the corresponding author upon reasonable request.
