# Supplementary material for: Intelligent Pyroptosis Inducer for Precise and Augmented Tumor Therapy Through Specific Activation Pyroptosis in Tumor
Source: Adv Sci (Weinh). 2024 Nov 27;12(3):2407713. doi: 10.1002/advs.202407713 (PMC11744558; doi:10.1002/advs.202407713)
Supplement: Supplementary file 1 — Supporting Information [file ADVS-12-2407713-s001.docx]

Supporting Information

Intelligent pyroptosis inducer for precise and augmented tumor therapy through specific activation pyroptosis in tumor

Linlin Huo, Shiqi Zhu, Muyao Li, Mingya Tan, Mengke Fan, Jiayi Zhao, Jie Zeng, Meiling Liu, Kunyan Liu, Chao Tong*, and Zhenghuan Zhao*

L. Huo, S. Zhu, M. Tan, M. Fan, J. Zhao, J. Zeng, M. Liu, K. Liu, Prof. Z. Zhao

College of Basic Medical Sciences, Chongqing Medical University, Chongqing 400016, China

E-mail: roddirck@cqmu.edu.cn

Dr. M. Li

College of Life Sciences and Medicine, Chengdu University of Traditional Chinese Medicine, Chengdu 610075, China

Prof. C. Tong

National Clinical Research Center for Child Health and Disorders, Ministry of Education Key Laboratory of Child Development and Disorders, Children's Hospital of Chongqing Medical University, Chongqing 401122, China

E-mail: chaotong@hospital.cqmu.edu.cn

***Supplementary Experimental Section***

***Materials and Reagents:*** Hexadecane-1,2-Diol and dopamine were purchased from Sigma-Aldrich. Ruthenium Acetylacetonate (Ru(acac)_3_), 1,3-diphenylisobenzofuran (DPBF), methionine, and riboflavin were purchased from Adamas. Benzyl ether (99%) and oleylamine (approximate C18-content 80-90%) were purchased from Acros. Iron (III) acetylacetonate (Fe(acac)_3_), oleic acid (tech. 90%) and 1-Octadecene (90%) were purchased from Alfa Aesar. 1,2-distearoyl-sn-glycero-3-phosphoethanolamine-N-[amino (polyethylene glycol)-2000] (DSPE-PEG2000 amine) and 5,5'-dithiobis-(2-nitrobenzoic acid) (DTNB) were purchased from J&K scientific. Tetrahydrofuran, n-hexane, ethanol, and chloroform were purchased from Sinopharm Chemical Reagent Co. Ltd. (Shanghai, China). Methylene blue (MB), 2′,7′-dichlorodihydrofluoresceinn diacetate (DCFH-DA), and calcein acetoxymethyl ester (calcein AM)/propidium iodide (PI) apoptosis detection agents, and 5,5′,6,6′-tetrachloro-1,1′,3,3′-tetraethyl-imidacarbocyanine iodide (JC-1) staining kit were purchased from Dalian Meilun Biotechnology Co. Ltd. HKON-1r was purchased from MedChemExpress. ([Ru(dpp)_3_] Cl_2_(RDPP)) was purchased from Macklin. ATP assay kit was purchased from Beyotime Biotechnology. fluorescein (FITC)-conjugated affineur goat anti-rabbit IgG (H+L), FITC plus anti-mouse CD11c (N418) and APC anti-mouse CD 80 (B7-1) were purchased from Proteintech. PE anti-mouse CD86 (B7-2) was purchased from ThermoFisher. HMGB1, TNF-α, IFN-γ, IL-1β Elisa kits were purchased from Quanzhou Jiubang Biotechnology Co., Ltd.

***Characterizations:*** Transmission electron microscopy (TEM) and high-resolution TEM (HRTEM) were recorded using a JEM-2100 microscope with an accelerating voltage at 200 kV. The X-ray diffraction (XRD) patterns were obtained with X-ray powder diffraction on PANalytical X'Pert³ PRO. Field-dependent magnetization (*M-H*) curve curves were tested with Quantum Design PPMS-9. The X-ray photoelectron spectra (XPS) were obtained on Thermo Scientific K-Alpha+. Dynamic light scattering (DLS) measurements and zeta potential were conducted on a Brookhaven NanoBrook 90Plus PALS. The contents of Mn and Pt in particles were measured by inductively coupled plasma-optical emission spectroscopy (ICP-OES, Aglient 5110). The *T*_2_-weighted phantom images, *T*_2_ relaxation time and mice MR images measurements were performed on a 7 T MRI scanner (Bio-Spec, Bruker, Karlsruhe, Germany). The UV-vis absorption spectra were recorded from UV-vis spectrophotometer of Shimadzu UV-2600i. The NIR laser was produced using an 808 nm light-power laser (Hi-Tech Optoielectronics Co., Ltd). The electron spin resonance (ESR) spectrometer was measured by a Bruker A300-10/12. The flow cytometry assays were conducted on a FACS Vantage SE.

***Synthesis of SIRPI:*** 350 mg of Fe(acac)_3_, 398 mg of Ru(acac)_3_, 1.6 g of 1,2-hexadecanediol, 2 mL oleylamine and 2 mL oleic acid were dissolved in 15 mL of 1-Octadecene. The mixture was degassed in vacuum for 5 min and heated to 90 °C for 15 min under nitrogen environment to remove any low volatile impurities. Subsequently, the reaction mixture was heated to reflux at 200 °C for 45 min until the powder completely dissolved, the solution appears wine red color. The reaction solution was further heated to 280 ° C and keep it for 30 min. After cooling to room temperature, the product was collected and stored in 15 mL tetrahydrofuran. Then, SIRPI was modified with DSPE-PEG-NH_2_ to enhance the biocompatibility. Briefly, SIRPI (200 μL) and 2 mg of DSPE-PEG-NH_2_ were dispersed in 240 μL chloroform and sonicated to ensure them well mixed. After chloroform totally removed, 200 μL of deionized water was added and sonicated again to obtain a uniform monodisperse solution of SIRPI.

***Photothermal Performance of SIRPI:*** 1 mL of SIRPI aqueous solutions with different concentrations (0-200 μg/mL) were irradiated with 808 nm laser, the temperatures were recorded by a thermocouple probe.

***Superoxide Anions (O_2_^.-^) Scavenging Activity of SIRPI:*** Methionine (130 mM,120 μL), riboflavin (200 μM, 120 μL), NBT (750 μM ,120 μL), and SIRPI with final concentrations of 0, 4.6, 9.2, 18.4, 36.7 μg/mL were added to PBS solution (0.01 M, pH 7.4) and fixed the final volume to 1.2 mL, respectively. Then the mixed solutions were irradiated with a 365 nm UV lamp for 15 min. Following, the absorbance at 560 nm was measured immediately by a microplate reader. The elimination percentage was calculated as follows: elimination rate (%) = [(A_0_-A)/A_0_] × 100%, where A is the absorbance of each sample and A_0_ is the absorbance of the control.

***Singlet Oxygen (^1^O_2_) Scavenging Activity of SIRPI Evaluated by ESR Measurement:*** SIRPI (25 μg/mL and 50 μg/mL) and TEMP were added to H_2_O to a final volume of 2 mL. Then the mixed solutions were placed in a dark box and irradiated with a 660 nm laser at 51 mW/cm^2^ for 5 min, and detected by using EPR. TEMP was used as control. H_2_O_2_ (7.5 mM, 200 μL) and sodium hypochlorite (2.5 mM, 200 μL) were incubated for 5 min to generate ^1^O_2_. Subsequently, SIRPI (500 μg/mL) and deionized water we added to a total volume of1.6 mL and incubated for another 5 min. The residue ^1^O_2_ was traped by TEMP, and the ESR spectra were recorded by Bruker A300-10/12 sepctrometer.

***Hydroxyl Radical (·OH) Scavenging Activity of SIRPI:*** FeSO_4_ (1.8 mM, 0.35 mL) was mixed with H_2_O_2_ (5 mM, 0.35 mL) and stirred at room temperature for 10 min. Then SIRPI with final concentrations of 0, 12.5, 25, 50 μg/mL were added to the mixed solution and stirred for 5 min. Finally, Methylene blue (MB, 100 μg/mL) was added to the mixed solution and stirred for 30 min. After the reaction was finished, the absorbance at 665 nm was measured immediately with an microplate reader. The elimination rate was calculated as follows: elimination rate (%) = [(A_0_-A)/A_0_] × 100%, where A is the absorbance of each sample and A_0_ is the control absorbance.

***CAT-like Activity of SIRPI:*** Brifly, H_2_O_2_ (30%, 5 µL) and SIRPI (1 mg/mL, 100 μL) were added to 10 mL of deionized water under vigorous stirring in vacuum. Then, the generated concentration of O_2_ was monitored by a dissolved oxygen meter (ST300D, OHAUS).

***Computational Method of Density Functional Theory (DFT) :*** We used the DFT as implemented in the Vienna Ab initio simulation package (VASP) in all calculations. The exchange-correlation potential is described by using the generalized gradient approximation of Perdew-Burke-Ernzerhof (GGA-PBE). The projector augmented-wave (PAW) method is employed to treat interactions between ion cores and valence electrons. The plane-wave cutoff energy was fixed to 400 eV. Given structural models were relaxed until the Hellmann–Feynman forces smaller than -0.02 eV/Å and the change in energy smaller than 10^-5^ eV was attained.

***Singlet Oxygen (^1^O_2_) Generation of SIRPI:*** We used the dithiobis-(2-nitrobenzoic acid) (DPBF) as an indicator. Briefly, SIRPI (500 μg/mL, 300 μL) with or without H_2_O_2_ (100 mM, 75 μL) were added to water containing DPBF (1 mg/mL, 30 μL), and incubated with or without 808 nm laser irradiation (1W/cm^2^) for 10 min. Then centrifuged to remove SIRPI, and the absorbance of DPBF at 415 nm was recored by UV-vis spectrophotometer.

***Singlet Oxygen (^1^O_2_) Generation of SIRPI Evaluated by ESR Measurement:*** SIRPI (500 μg/mL, 30 μL) with or without H_2_O_2_ (100 mM, 10 μL) was added to PBS buffer, adding triethylamine hydrochloride (TEMP), and irradiated with or without 808 nm laser irradiation (1W/cm^2^) for 5 min. The ESR spectra was recorded by Bruker A300-10/12 sepctrometer.

***Hydroxyl Radical (·OH) Generation of SIRPI:*** We used the methylene blue (MB) as an indicator. Briefly, SIRPI (2.5 mg/mL, 50 μL) was added to PBS, and incubated at 37 °C and stirred for 1 hour. Then centrifuged and samplied 200 μL of supernatant, then H_2_O_2_ (100 mM, 100 μL) and MB (100 μg/mL, 100 μL) were added sequentially and incubate at 37 ℃ for 30 min. UV-vis spectrophotometer was used to record the absorbance of MB at 665 nm.

***Hydroxyl Radical (·OH) Generation of SIRPI Evaluated by ESR Measurement:*** SIRPI (500 μg/mL, 30 μL) was added to PBS, and incubated at 37 °C or 808 nm laser irradiation (1W/cm^2^) for 5 min. Then centrifuged and samplied 200 μL of supernatant, H_2_O_2_ (100 mM, 20 μL), and trapping agent 5, 5-dimethyl-1-pyrrolineN-oxide (DMPO) were added sequentially and incubate at 37 ℃ or 808 nm laser irradiation (1 W/cm^2^) for 5 min. The ESR spectra was recorded by Bruker A300-10/12 sepctrometer.

***In Vitro MR imaging:*** SIRPI with different concentration (Fe:0, 0.1, 0.2, 0.4, 0.8, 1.6 mM) were scanned by a 3 T MAGNETOM Prisma MRI scanner. The following parameters were used to obtain *T*_2_ value: echo time (TE) = 80.0 ms; repetition time (TR) = 4000.0 ms; thickness =4.0 mm; field of view (FOV) = 200.0 × 200.0 mm^2^; slice =5; matrix = 128 × 128.

***Cell Culture:*** Murine breast cancer (4T1) cells, human breast cancer (MCF-7) cells and Hs578bst cells were purchased from Cell Bank of Chinese Academy of Sciences and Shanghai Jinyuan Biological respectively. They were culture at 37 ℃ under 5% CO_2_ atmosphere in medium RPMI 1640 and supplemented with 10% fetal bovine serum (FBS) and 1% penicillin /streptomycin. To create the hypoxic and normoxic condition, the oxygen content of incubator was set as 1% and 20%, respectively.

***Cell Uptake:*** 4T1, MCF-7 and Hs578bst cells were seeded in the 6-well plate at a density of 2×10^5^ cells per well and incubated for 24 h. Then the medium was extracted and the cells were washed with the PBS, followed by addition of Rhodamine B-labeled SIRPI (200 μM). After 8 h, the cell uptake was monitored by a fluorescent microscopy.

***Escape from Lysosomal:*** 4T1 cells were seeded in the confocal dish at a density of 1×10^5^ cells and incubated for 24 h. Then the medium was removed and the cells were washed with the PBS, followed by addition of Rhodamine B-labeled SIRPI (200 μM) and incubated for 2 and 6 h, respectively. Subsequently, the medium was removed and stained sequentially with LysoTracker Green DND-26 for 30 min and Hoechst 33342 for 30 min. The fluorescence intensities of Rhodamine B, LysoTracker Green DND-26, and Hoechst 33342 were monitored by confocal laser scanning microscope (Leica SP8)..

***Cytotoxicity in vitro:*** 4T1, MCF-7 and Hs578bst cells were seeded in 96-well plate at a density of 1×10^4^ cells per well for 24 h under hypoxic or normoxic condition to allow cell attachment. Subsequently, the medium was removed and the cells were washed with PBS, and then fresh complete medium containing SIRPI was added. After incubation for 24 h, the cells were washed with PBS, and 10 μL of CCK-8 and 100 μL of fresh RPMI-1640 was added to each well and incubated for 1 hour. The absorbance of each well was measured by a microplate reader at 450 nm, and the cell viability was calculated.

***Inhibition Efficacy of SIRPI in vitro:*** The 4T1, MCF-7 and Hs578bst cells were seeded in 96-well plate with a density of 1×10^4^ cells per well for 24 h in hypoxic or normoxic conditions. Then medium was abandoned, different concentration of SIRPI and H_2_O_2_ (50 μM) dispersed in medium were added into 96-well plates with the cells, and incubated for another 20 h. Then the cells were irradiated with an 808 nm NIR Laser (1W/cm^2^) for 5 min. After laser irradiation, the cells were incubated for another 4 h. Finally, the CCK-8 assay was used to measure the cell viability, respectively.

***Calcium fluorescein (AM) and PI staining:*** The 4T1 cells and Hs578bst cells were seeded in 96-well plate and incubated overnight in hypoxic or normoxic condition, followed by treated with SIRPI (200 μM or 300 μM) and H_2_O_2_ (50 μM). After 8 h, the cells were irradiated by the 808 nm NIR Laser(1W/cm^2^) for 5 min. Then the cells were incubated for 4 h and stained with both Calcein-AM and PI for 15 min. The fluorescence intensities were monitored with a fluorescent microscopy.

***ROS Generation in vitro:*** 4T1 and Hs578bst cells were seeded in the 96-well plate at a density of 1.5 × 10^4^ cells per well and incubated overnight under hypoxic or normoxic condition. SIRPI (200 μM or 300 μM) and H_2_O_2_ (50 μM) dispersed in medium were added to the cells and incubated for 7.5 h. Then the cells were irradiated with an 808 nm NIR Laser (1 W/cm^2^) for 5 min, washed with PBS, and the DCFH-DA (10 μM) was incubated with cells for 30 min. Then, cells were washed with PBS, the DCF fluorescence was observed by using fluorescence microscopy.

***•OH Generation in vitro:*** The 4T1 and Hs578bst cells were seeded in the 96-well plate at a density of 1.5× 10^4^ cells per well and incubated overnight under hypoxic or normoxic condition. SIRPI (200 μM or 300 μM) and H_2_O_2_ (50 μM) dispersed in medium were added into the cells and incubated for 7.5 h. Then the cells were irradiated with 808 nm NIR Laser (1 W/cm^2^) for 5 min, washed with PBS, and the HKOH-1r (10 μM) was incubated with cells for 30 min, cells were washed with PBS, the HKOH-1r fluorescence was observed on the fluorescence microscopy.

***^1^O_2_ Generation in vitro:*** 4T1 and Hs578bst cells were seeded in the 96-well plate at a density of 1.5× 10^4^ cells per well and incubated overnight under hypoxic or normoxic condition. SIRPI (200 μM or 300 μM) and H_2_O_2_ (50 μM) dispersed in medium were added into the cells and incubated for 7.5 h. Then the cells were irradiated with an 808 nm NIR Laser (1 W/cm^2^) for 5 min, washed with PBS, and the SOSG (5 μM) was added and incubated with cells for 30 min, then, cells were washed with PBS, the fluorescence of SOSG was observed under fluorescence microscopy.

***O_2_ Generation in vitro:***The 4T1 cells were seeded in 96-well plates at a density of 1.5× 10^4^ cells per well and incubated in 37 ℃ with 5% CO_2_ and 1% O_2_ overnight. The culture medium was replaced by fresh medium containing 10 μg/mL [Ru(dpp)_3_] Cl_2_ (RDPP). After 6 h, the medium was removed, and the cells were washed with PBS. Then, SIRPI was added and co-incubated under the hypoxia condition for another 6 h, followed by 808 nm laser (1W/cm^2^) irradiation. Subsequently, cells were washed with PBS, the fluorescence images were acquired by fluorescent microscopy.

***ROS Scavenging Activity in vitro:.*** 4T1 cells and Hs578bst cells were seeded in the 96-well plate at a density of 1.5× 10^4^ cells per well. Rosup (50 μg/mL) was dispersed in RPMI 1640 medium and added to the cells for 2 h incubation at 37 ℃ to produce ROS. Subsequently the supernatants were removed and the cells were incubated with SIRPI (200 μM) in medium. After 4 h, we removed the medium again and added DCFH-DA 30 min incubation. The fluorescence of DCF was observed under fluorescence microscopy.

***Mitochondrial Function Measurement:*** 4T1 cells and Hs578bst cells were seeded in the 96-well plate at a density of 1.5× 10^4^ cells per well and incubated overnight under hypoxic or normoxic condition. Then the medium was extracted and the cells were washed with the PBS, followed by addition of SIRPI (200 μM or 300 μM) and H_2_O_2_ (50 μM). After 10 h, the cells were irradiation with the 808 nm NIR Laser (1 W/cm^2^) for 5 min and then incubated for 2 h. Then the treated cells were washed with PBS and stained with JC-1 for 30 min. Lastly, cells were washed with buffer solution, and fluorescent microscopy was performed to acquire the fluorescence images.,

***Danger-Associated Molecular Patterns (DAMPs) Detection in vitro:*** For extracellular HMGB1 detection, the 4T1 cells and Hs578bst cells were seeded in the 12-well plate at a density of 2.5× 10^5^ cells per well and incubated for 24 h under hypoxic or normoxic condition. Then the medium was extracted and the cells were washed with the PBS, followed by addition of SIRPI (400 μM). After 12 h, the cells were irradiated by the 808 nm NIR Laser (1 W/cm^2^) for 5 min and incubated for another 12 h, the treated cells supernatant was collected by centrifugation, respectively, and the release of HMGB1 in supernatant was measured by the by enzyme-linked immunosorbent assay (ELISA) Kit according to the manufacturer’s protocols. For ATP release detection. The 4T1 cells were seeded in the 6-well plate at a density of 2×10^5^ cells per well and incubated for 24 h. Then the medium was extracted and the cells were washed with the PBS, followed by addition of SIRPI (400 μM). After 12 h, the cells were irradiated by the 808 nm NIR Laser (1 W/cm^2^) for 5 min and incubated for another 12 h. Then the treated cells supernatant was collected by centrifugation, respectively, and the release of ATP in supernatant was measured by the Chemiluminescence ATP Determination Kit according to the manufacturer’s protocols. For extracellular LDH detection. 4T1 cells and Hs578bst cells were seeded in 96-well plate at a density of 1×10^4^ cells per well for 24 h under hypoxic or normoxic condition to allow cell attachment. Subsequently, the medium was removed and the cells were washed with PBS, and then fresh complete medium containing SIRPI was added. After incubated for 24 h and washed with PBS, the cells supernatant was collected, and the release of LDH in supernatant was measured by the LDH Release Assay Kit according to the manufacturer’s protocols.

***IL-1β Detection in vitro:*** For extracellular released IL-1β detection, the 4T1 cells were seeded in the 12-well plate at a density of 2.5× 10^5^ cells per well and incubated for 24 h under hypoxic or normoxic condition. Then the medium was extracted and the cells were washed with the PBS, followed by addition of SIRPI (400 μM). After 12 , the cells were irradiated by the 808 nm NIR Laser (1 W/cm^2^) for 5 min and incubated for another 12 h, the treated cells supernatant was collected by centrifugation, respectively, and the release of IL-1β in supernatant was measured by the by enzyme-linked immunosorbent assay (ELISA) Kit according to the manufacturer’s protocols.

***Cell Morphological Observation to Evaluate Specific Pyroptosisi in vitro:*** 4T1 cells and Hs578bst cells were seeded in the 96-well plate at a density of 1.5× 10^4^ cells per well and incubated overnight under hypoxic or normoxic condition. Then SIRPI (400 μM) was added in the wells and incubated for 12 h. Moreover, the cells were irradiation with the 808 nm NIR Laser (1 W/cm^2^) for 5 min, and analyzed after 12 h of incubation. Observing the cell morphology using a microscope (Leica dmilled fluo), the cells undergoing pyroptosis would show a remarkable swelling like blowing bubbles.

***Western Immunoblot (WB) of Specific Pyroptosis in vitro:*** Briefly, the 4T1 cells and Hs578bst cells were seeded in the 6-well plate at a density of 2×10^5^ cells per well and incubated for 24 h under hypoxic or normoxic condition. Then the medium was extracted and the cells were washed with the PBS, followed by addition of SIRPI (400 μM). After 12 h, the cells were irradiated by the 808 nm NIR Laser (1 W/cm^2^) for 5 min and incubated for another 12 h, the cells were split with RIPA lysis buffer for 30 min and centrifugated (12000 g, 5 min) at 4 °C. Then we used the BCA protein kit (Dalian Meilun Biotechnology Co. Ltd.) to measure the protein concentration of the supernatant. The acquired protein was separated by 10% SDS-PAGE gel electrophoresis and transferred onto PVDF membranes. The PVDF membranes were examined by using Chemiluminescence imaging system (Guangzhou Light Instrument Biotechnology Co. LTD, OI-600) after incubation with primary antibody for 12 h at 4 °C and horseradish peroxidase-labelled secondary antibody. The antibodies used in this study were as follows: cleaved Caspase-3 (#9664, cell signaling technology, CST), GSDME (ab215191, Abcam, Cambridge, UK).

***Apoptosis Assay.*** Briefly, the 4T1 cells were seeded in the 6-well plate at a density of 2×10^5^ cells per well and incubated for 24 h. Then the medium was extracted and the cells were washed with the PBS, followed by addition of SIRPI (300 μM). After 12 h, the cells were irradiated by the 808 nm NIR Laser (1 W/cm^2^) for 5 min and incubated for another 12 h, followed by quantified by an annexin V-FITC/PI apoptosis detection kit using an FACS Vantage SE flow cytometer.

***RNA Sequencing Measurement:*** the 4T1 cells and Hs578bst cells were seeded in the 12-well plate at a density of 2.5× 10^5^ cells per well and incubated for 24 h under hypoxic or normoxic condition. Then the medium was extracted and the cells were washed with the PBS, followed by addition of SIRPI (200 μM). After 12 h, the cells were irradiated by the 808 nm NIR Laser (1 W/cm^2^) for 5 min and incubated for another 12 h. RNA-seq was conducted on Nova Seq 6000 platform (Beijing, China).

***Hemolysis Analysis:*** 1 mL blood was collected from healthy Balb/c mice, after centrifugating and washing with PBS solution for four times, 80 μL red blood cells (RBCs) were added to 800 μL deionized water, PBS, or SIRPI (0,10, 20, 40, 60, 80, 100, 200 μM) diapered in PBS for 2 h incubation at 37 ℃, respectively. The RBCs in PBS was used as a negative control, and the RBCs in deionized water was used as a positive control. Following centrifugation, the absorbance of the supernatant at 540 nm was measured using an enzyme-linked immunosorbent (Thermo Multiskan FC) and the hemolysis ratio was calculated using the formula:(A_S_-A_N_)/(A_P_-A_N_) ×100%, where A_S_ is the absorbance of each sample, A_N_ is the absorbance of negative control absorbance and the A_P_ is the absorbance of positive control absorbance.

***Blood Biochemistry Analysis:*** The Balb/c mice were intravenously injected with saline and SIRPI (Fe: 2.5 mg/kg) for 24 h, then the blood was collected to measure the levels of liver function makers (alanine aminotransferase, aspartate aminotransferase, and alkaline phosphatase), and kidney function markers (creatinine, nephric blood urea, and uric acid).

***Tumor Model:*** The animal experiment procedures were conducted following an approved protocol by Institutional Animal Care and Use Committee of Chongqing Medical University. Female Balb/c mice were purchased from Chongqing Medical University. To create the tumor model, the 4T1 cell suspension (about 1×10^6^) was subcutaneously injected into the upper part of the right side back of the mouse. When the tumor grew about 75-100 mm^3^, the vivo imaging and anticancer examination were performed.

***MR Imaging in vivo:*** 4T1 bearing Balb/c mice were intravenously injected with 100 μL SIRPI (Fe: 2.5 mg/kg), The *T*_2_-wighted MR images were obtained at pre-injection,1 h post-injection. The parameters were used as follows: TE = 35.0 ms; TR = 2225.0 ms; thickness =0.8 mm; FOV = 35.0 × 30.0 mm^2^; slice = 18, image size = 200 × 200. For comparing the *T*_2_ imaging performance of the agents, we quantified and analyzed the imaging signal to obtain ΔSNR with the following equation: ΔSNR=(SNR_post_−SNR_pre_)/SNR_pre_×100% (SNR=SI/SD), where SI represents signal intensity in region of interest and SD represents standard deviation in MR images.

***Tumor Therapy in vivo:*** 4T1 bearing Balb/c mice were randomly divided into 4 groups with different treatments: Saline, Laser, SIRPI, SIRPI + Laser group, 100 μL saline and SIRPI with 2.5 mg/kg concentration of Fe ions was injected into tumor-bearing Balb/c mice via tail vein, respectively. After 1 h injection, the NIR laser (808 nm, 1 W/cm^2^) was applied to the tumor for 5 min. and the temperature changes were recorded by an infrared camera. The tumor growth and body weight changes of mice monitored every 2 days until day 14.

***Histology Analysis in vivo:*** After 14 days’ treatment, all the 4 groups of mice were sacrificed, and tumors were collected, weighted, and photographed. The tissues (heart, liver, spleen, lung, kidneys) and tumors were fixed using 4% paraformaldehyde solution. The tissues were processed with paraffin embedding routinely followed by H&E staining, terminal deoxynucleotidyl transferase-mediated dUTP nick-end labeling (TUNEL) staining and Ki67 staining.

***Western Immunoblot (WB) of Specific Pyroptosis in vivo:*** Tumor-bearing Balb/c mice with tumor size of about 75-100 mm were randomly divided into four groups, Saline, Saline+Laser, SIRPI, and SIRPI+Laser. SIRPI (Fe: 2 mg/kg) was injected into mice via tail vein. After 1 hour injection, the NIR laser (808 nm, 0.5 W/cm^2^) was applied to the tumor for 5 min. Later, tumor tissues and normal tissues were lysed with RIPA buffer, grinded sufficiently, and ultrasonically for 30 min, followed by centrifugation (12000 g, 5 min) at 4°C to obtain proteins. Concentration of supernatant proteins was then measure using the BCA protein kit (Dalian Meilun Biotechnology Co. Ltd.), and acquired protein was separated by 10% SDS-PAGE gel electrophoresis and transferred onto PVDF membranes. The PVDF membranes were examined by using Chemiluminescence imaging system (Guangzhou Light Instrument Biotechnology Co. LTD, OI-600) after incubation with primary antibody for 12 h at 4 °C and horseradish peroxidase-labelled secondary antibody. The antibodies used in this study were as follows: cleaved caspase-3 (#9664, CST), GSDME (ab215191, Abcam), HIF-l alpha (ab179483, Abcam).

***Flow Cytometry of Immune Response in vivo:*** Spleens were acquired from 4T1 tumor-bearing mice after three times of treatments, and the single cell suspensions were prepared to analyze the maturation of dendritic cells (DCs) by flow cytometry. Briefly, the spleen tissue was gently grinded, and centrifugated to extracted the single cell suspension, subsequently, incubated the cells with FITC anti-CD11c, APC anti-CD80 and PE anti-CD86 antibodies. Flow cytometry was performed with an FACS Vantage SE flow cytometer, and all the samples were analyzed using FlowJo.

***Antitumor Immunity Effect of SIRPI in vivo:*** The differently treated tumor-bearing mice were sacrificed after 24 h treatment, collected blood and spleen tissue. The blood was used to extract serum for detecting the levels of TNF-α, IFN-γ, and IL-1β using Elisa kit according to the manufacturer’s protocols.

***Immunogenic to Inhibiter Lung Metastasis:*** Tumor-bearing Balb/c mice with tumor size of about 75-100 mm were randomly divided into four groups, Saline, Saline+Laser, SIRPI, and SIRPI+Laser. On days 0, 3 and 6, mice were injected with SIRPI (Fe: 2.5 mg /kg) via tail vein and the NIR laser (808 nm, 0.5 W/cm^2^) irradiation for 5 min to stimulate immune response *in vivo*. At day 7, mice were injected with 4×10^5^ 4T1 cells via tail vein of the mice, at day 23, the mice were sacrificed and the lung tissues were extracted to count the number of surface metastases and to record the number of internal metastases by H&E staining.


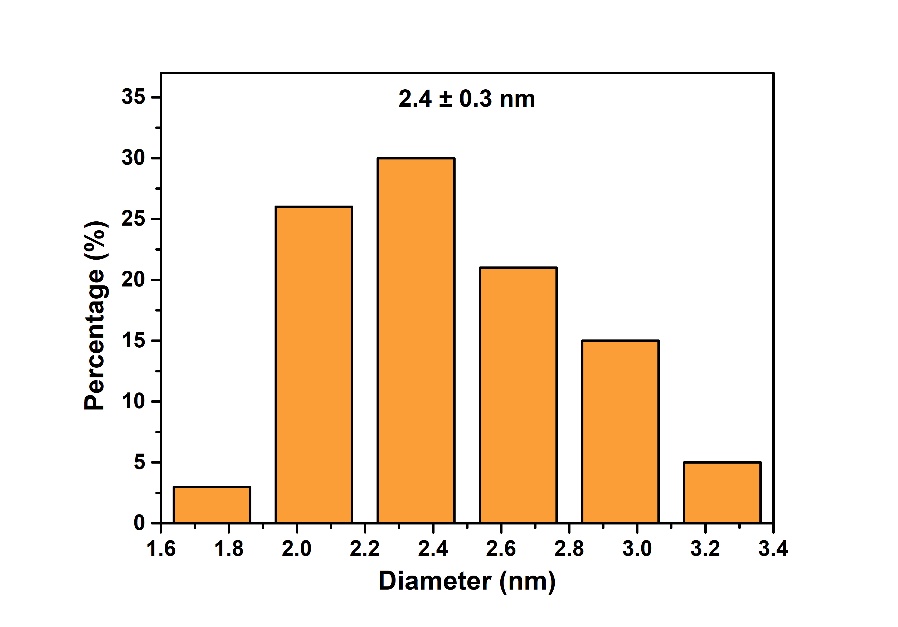


**Figure S1.** Particle size distribution of SIRPI.


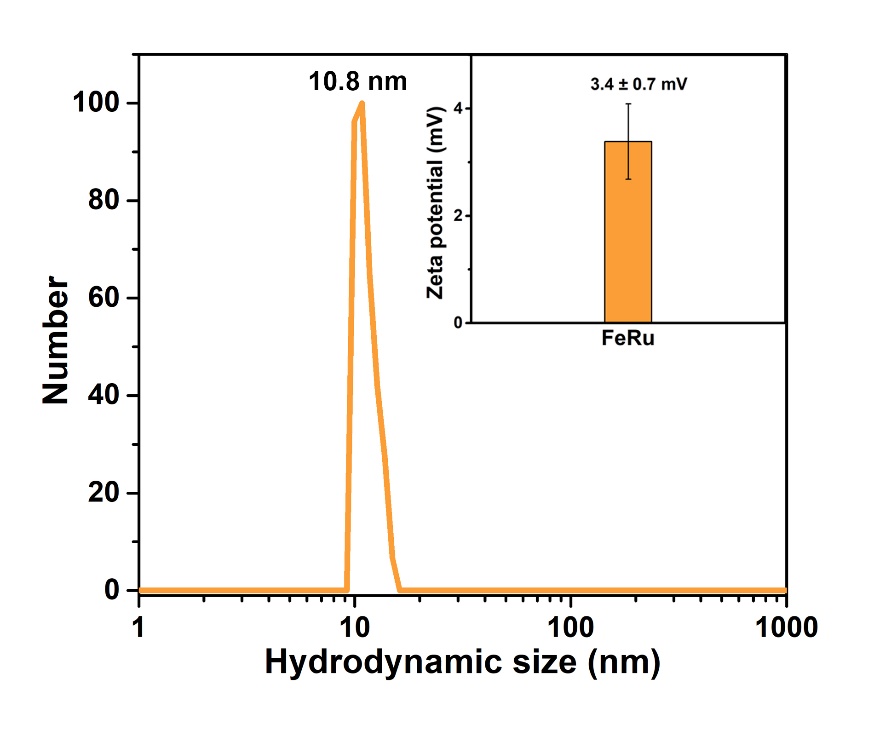


**Figure S2.** The hydrodynamic size distribution and zeta potential of SIRPI.


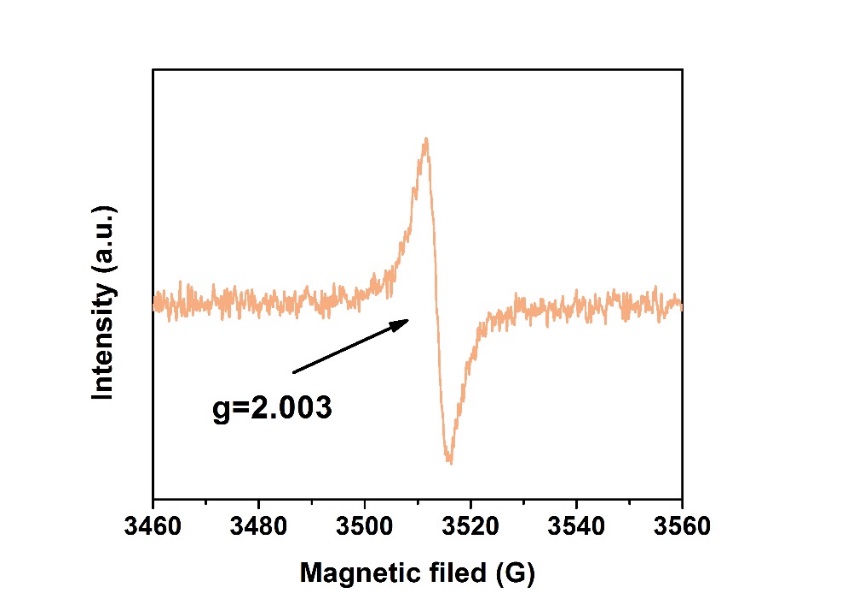


**Figure S3.** The oxygen vacancy of SIRPI.

**
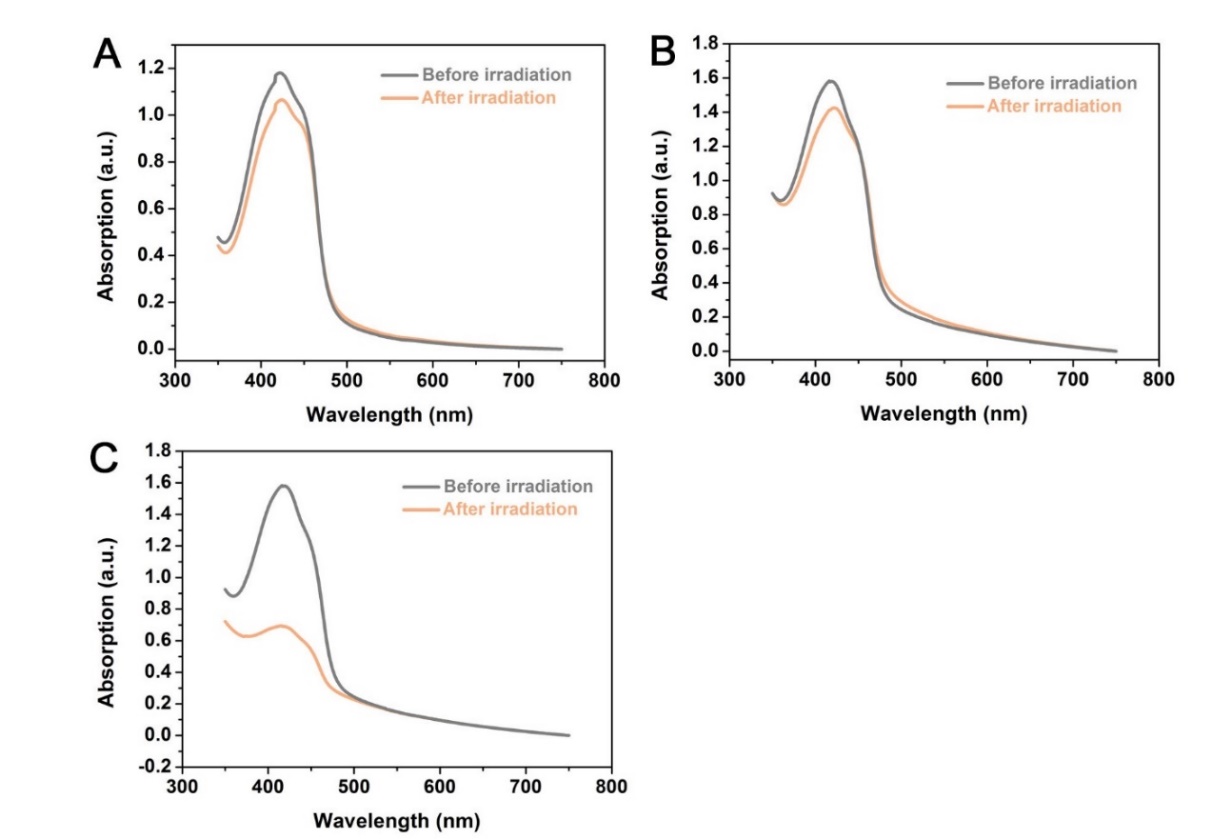
**

**Figure S4.** The UV-vis spectra of ^1^O_2_ generation with (A) H_2_O_2_+Laser, (B) SIRPI+Laser, (C) SIRPI+H_2_O_2_+Laser.


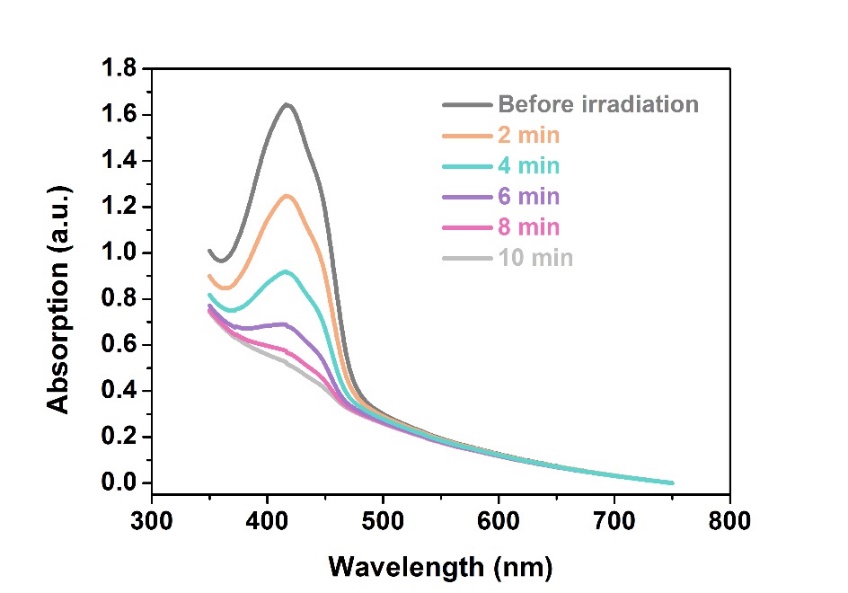


**Figure S5.** The UV-vis spectra of ^1^O_2_ generation of SIRPI+H_2_O_2_ with different times of NIR laser irradiation.


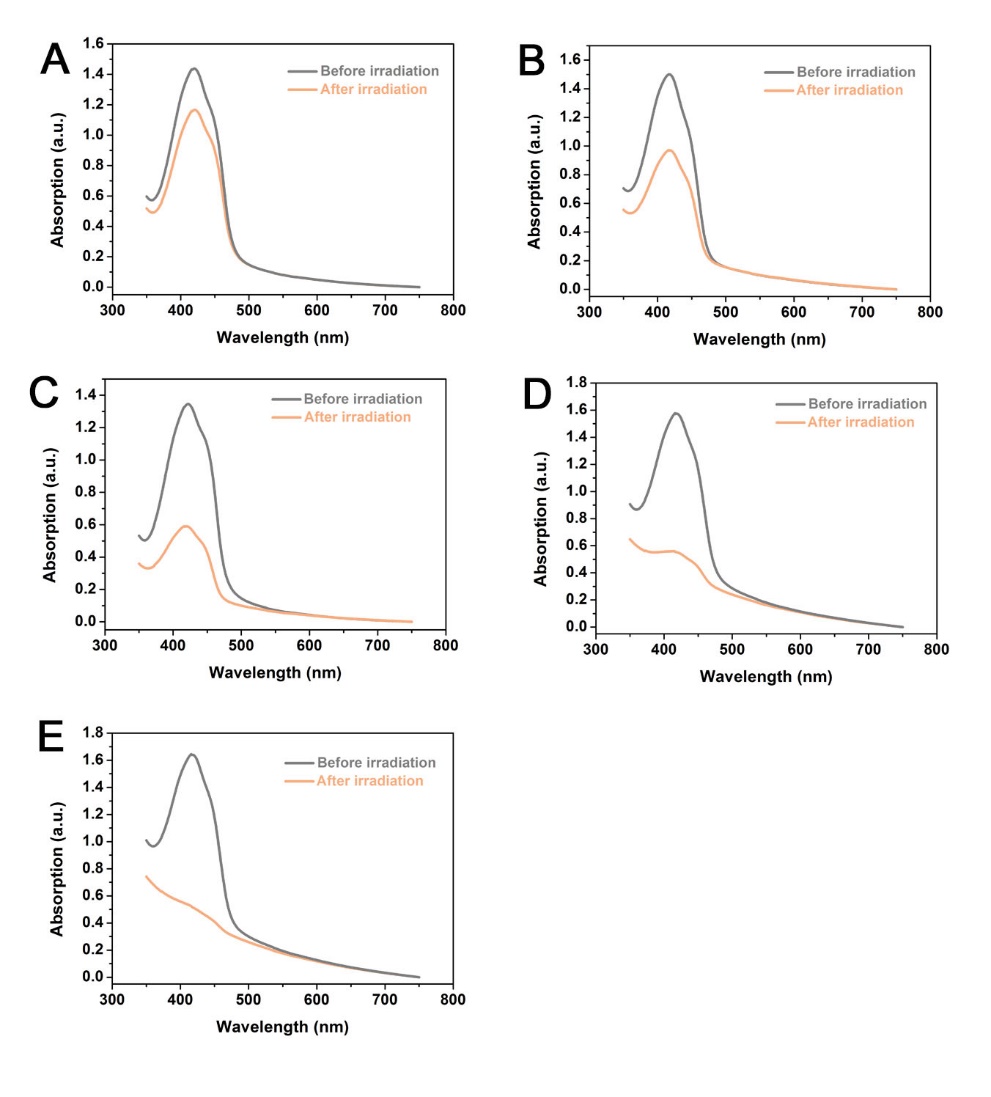


**Figure S6.** The UV-vis spectra of ^1^O_2_ generation with (A) 0 μg/mL, (B) 0.91 μg/mL, (C) 1.82 μg/mL, (D) 3.64 μg/mL, (E) 5.47 μg/mL of Ru in SIRPI and H_2_O_2_ before and after NIR laser irradiation.


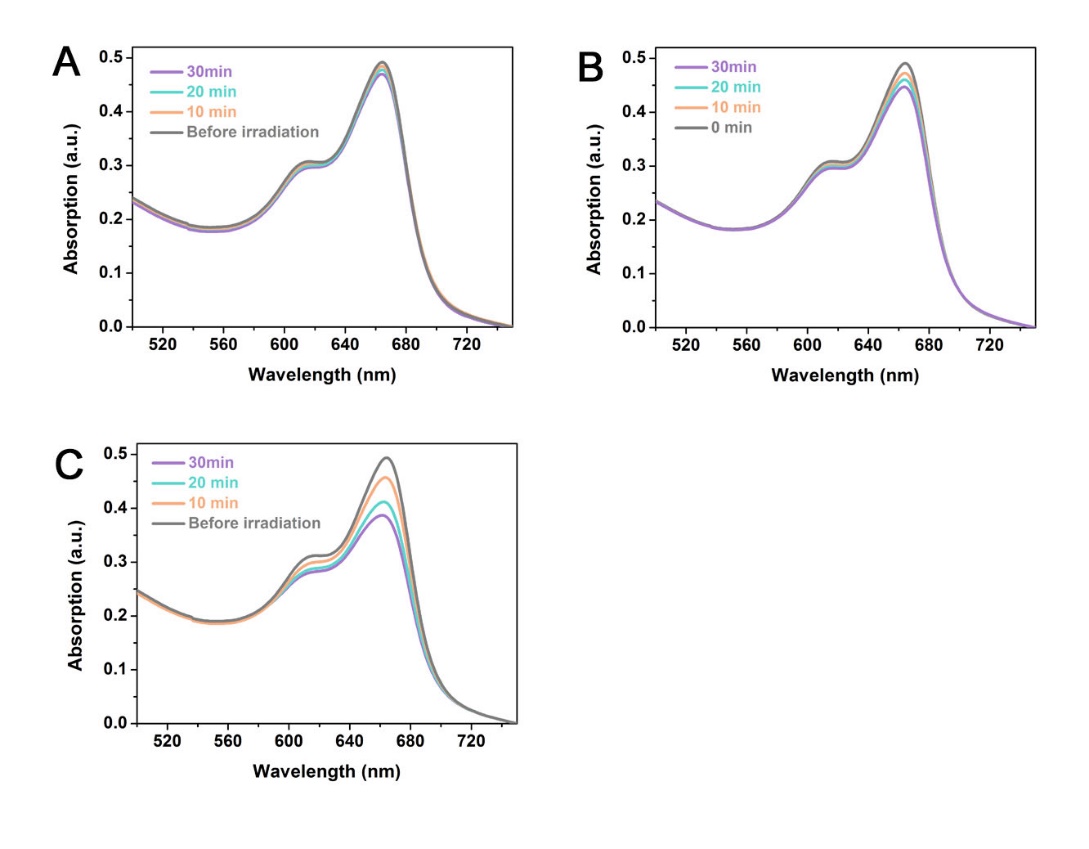


**Figure S7**. The UV-vis spectra of ·OH generation with (A) SIRPI+Laser, (B) SIRPI+H_2_O_2_, (C) SIRPI+Laser+H_2_O_2_.


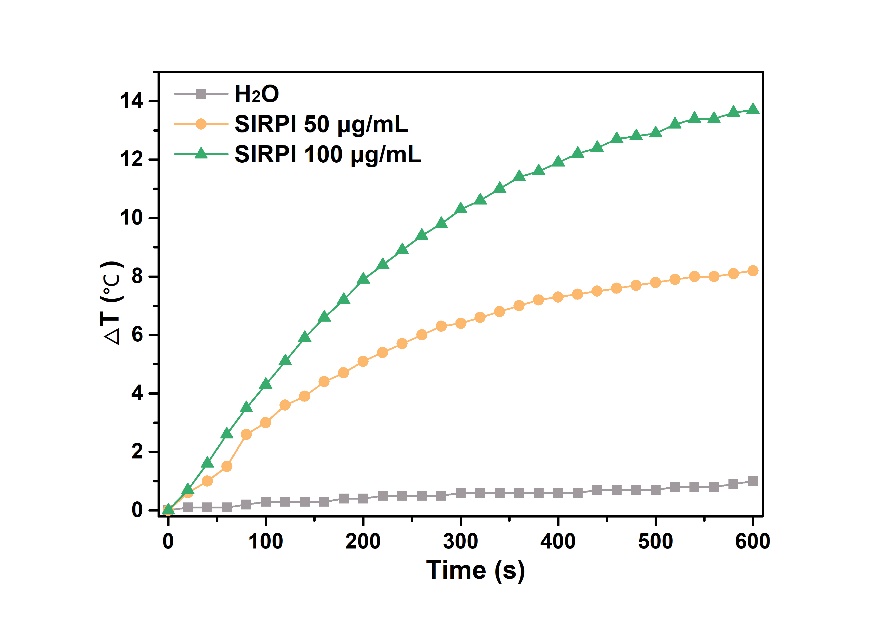


**Figure S8.** Temperature changes solution with different concentration of SIRPI (0-100 μg/mL) under NIR laser irradiation (1 W/cm^2^).


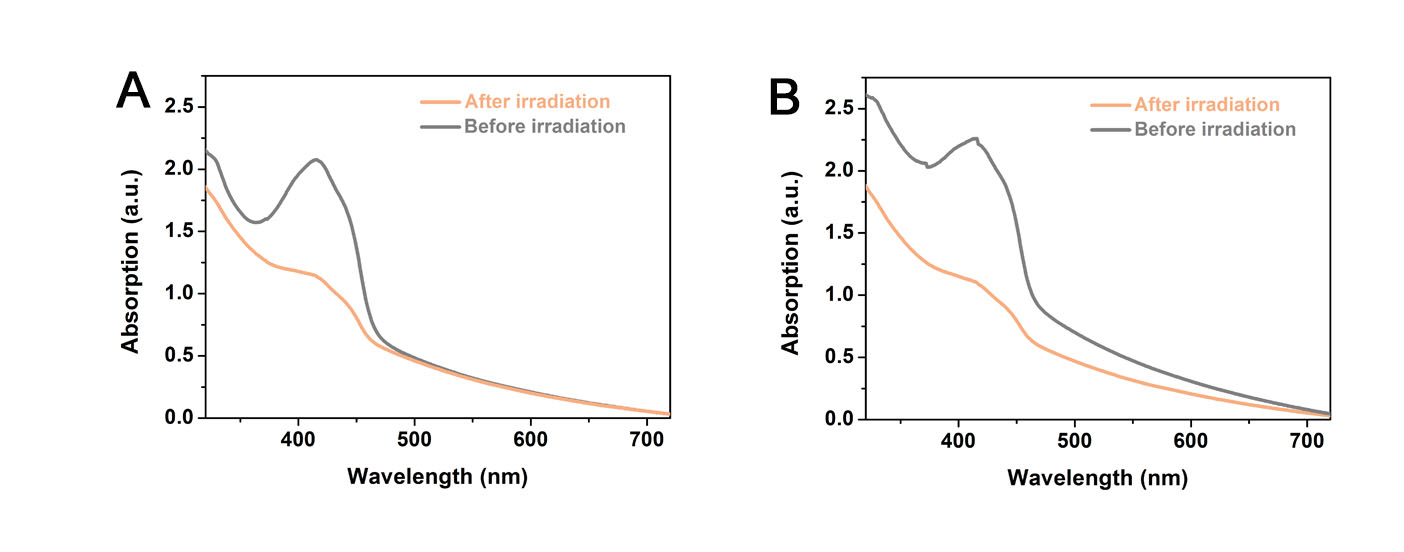


**Figure S9.** The UV-vis spectra of ^1^O_2_ generation by SIRPI under (A) normoxia (B) hypoxia condition.


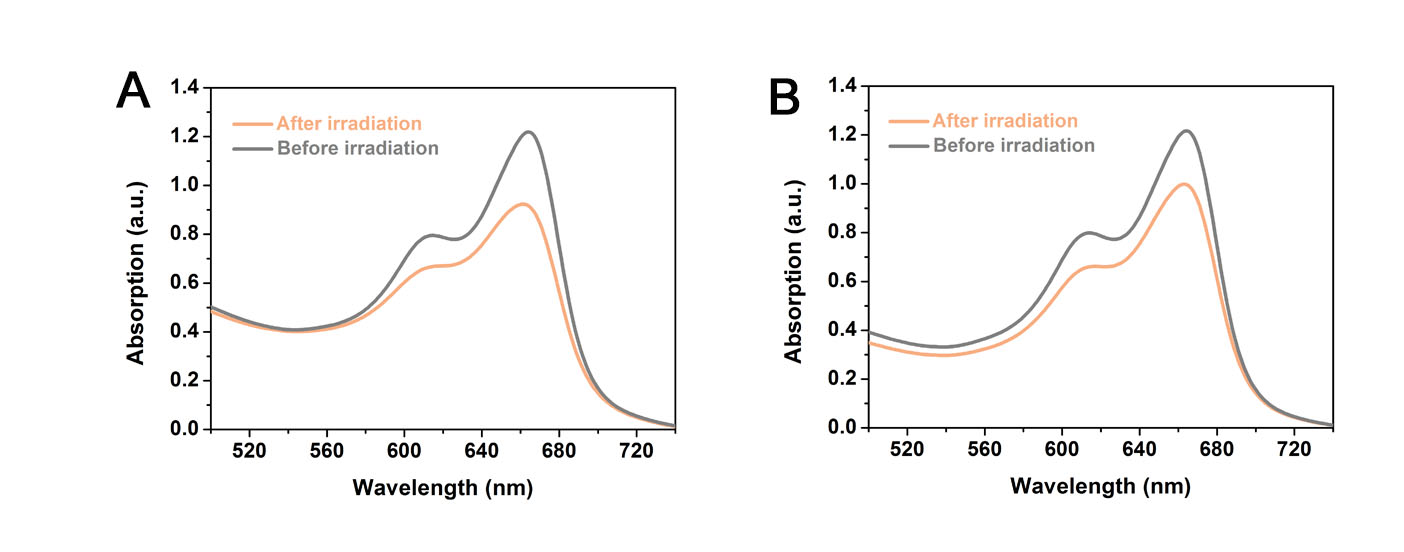


**Figure S10.** The UV-vis spectra of **·**OH generation by SIRPI under (A) normoxia and (B) hypoxia condition.


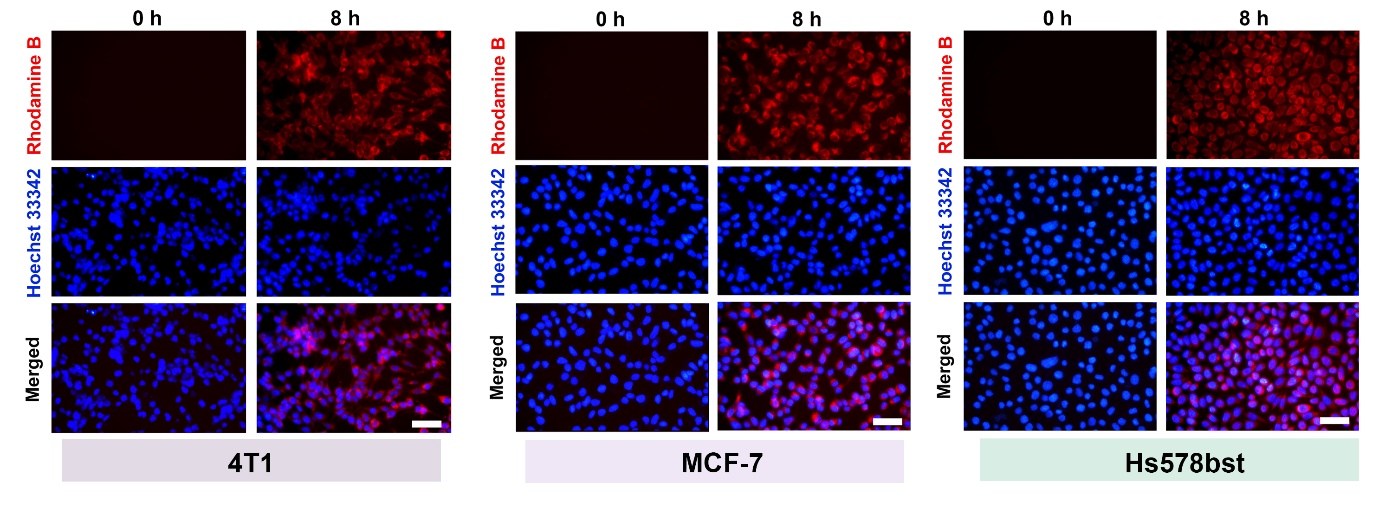


**Figure S11.** Fluorescence image of the uptake in 4T1, MCF-7, and Hs578bst cells, the scale bar is 50 μm.


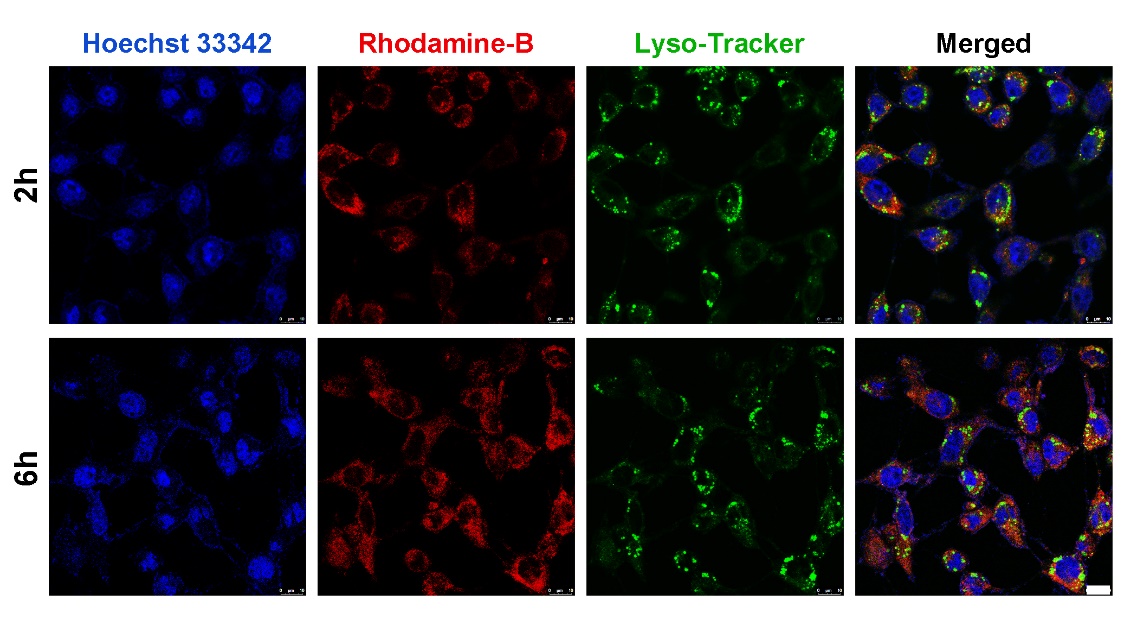


**Figure S12.** Colocalization of Rhodamine B-labeled SIRPI (red) with LysoTracker (green) in 4T1 cells after incubation for 2 and 6 h, the scale bar is 10 μm.


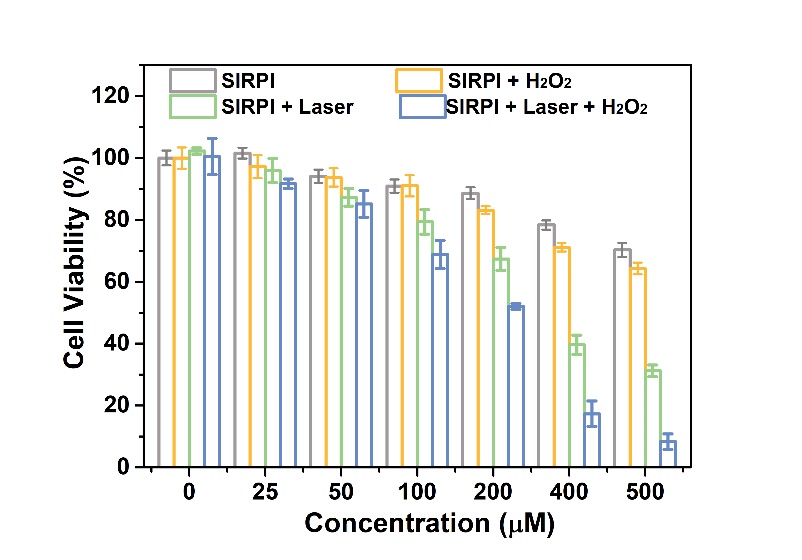


**Figure S13.** The relative viability of MCF-7 cells with different treatments (*n* = 3).


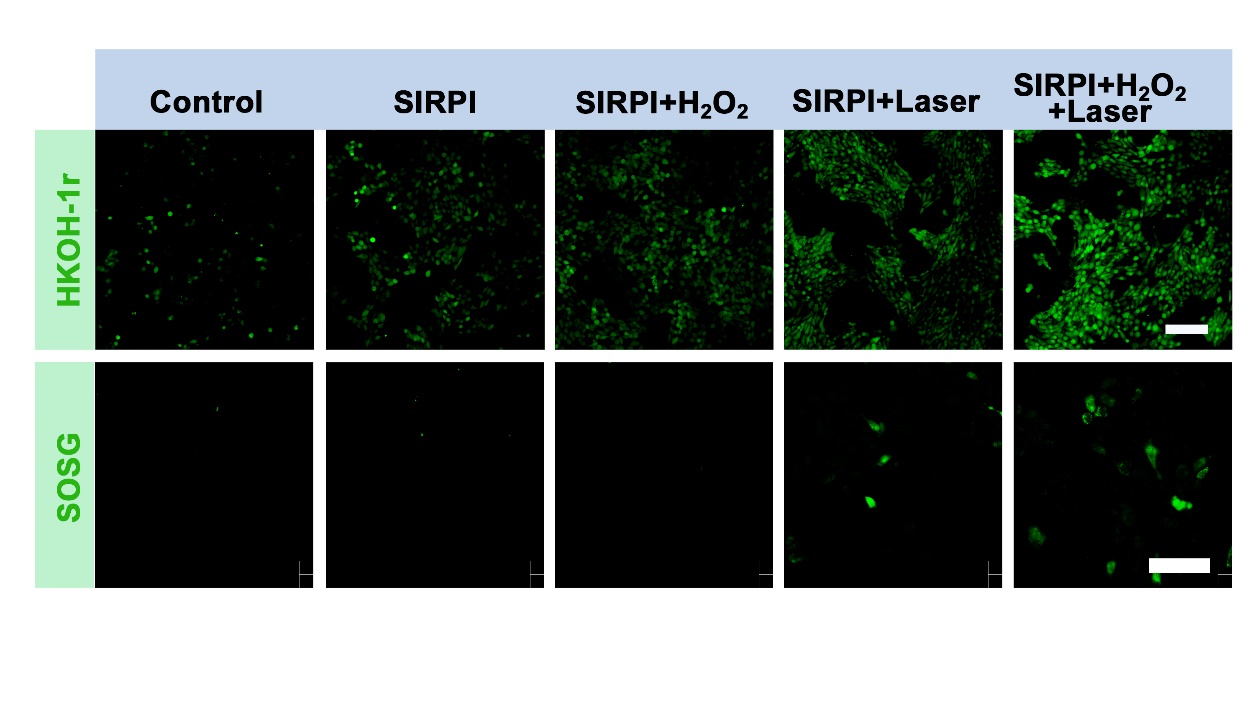


**Figure S14.** Fluorescence images of 4T1 cells treated differently to detect **·**OH (HKOH-1r as probe), and ^1^O_2_ (SOSG as probe) generation in normoxia incubation, the scale bar is 100 μm.


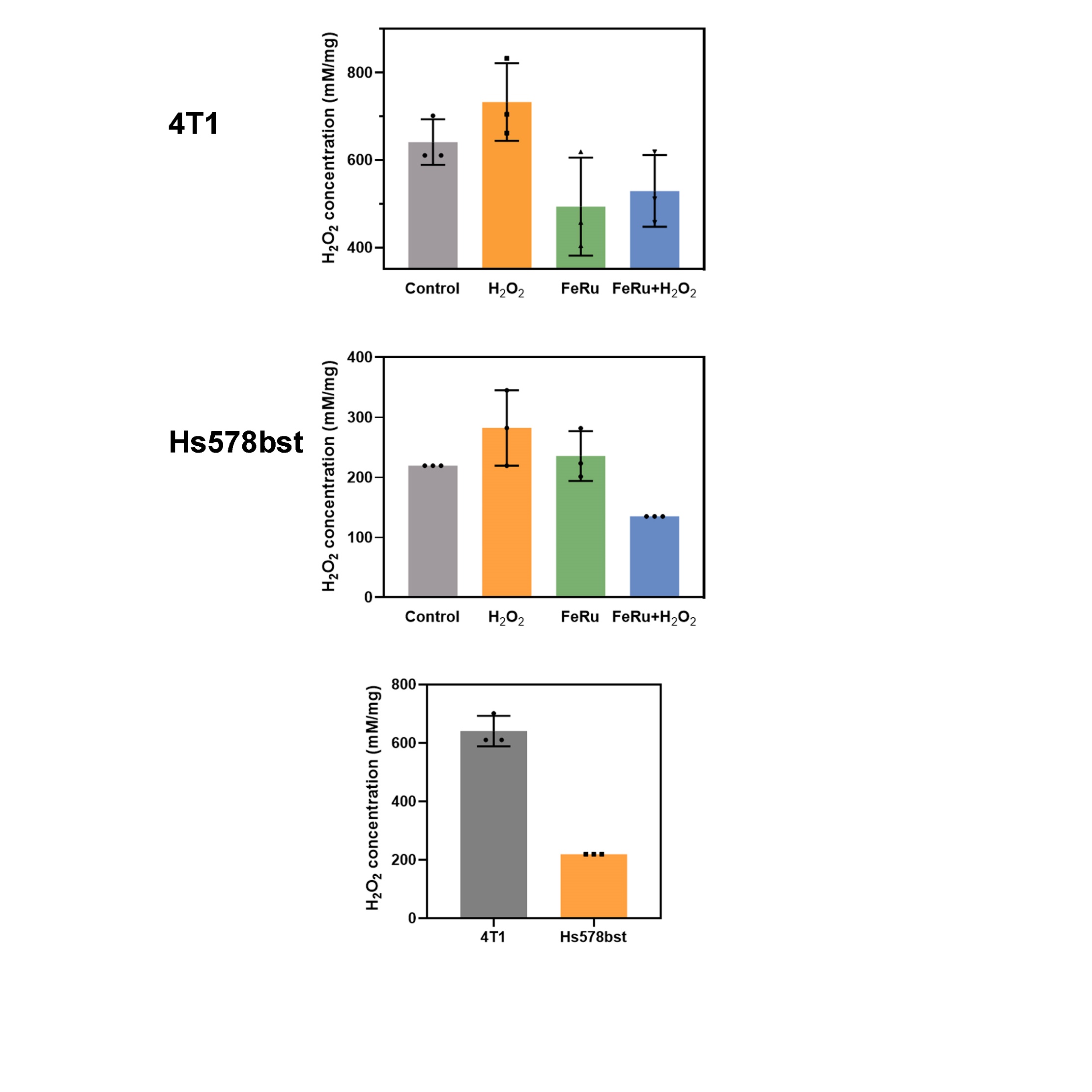


**Figure S15.** The H_2_O_2_ content in 4T1 cells and Hs578bst cells.


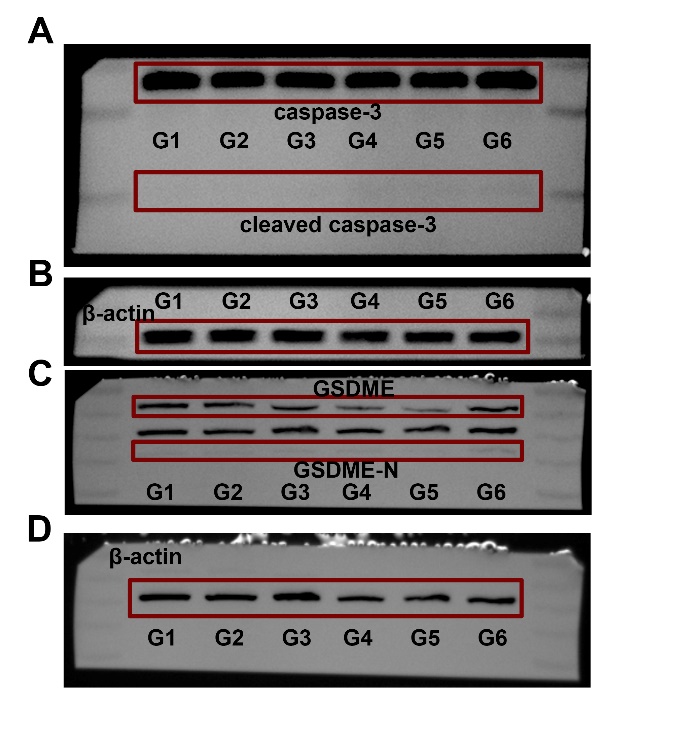


**Figure S16.** WB analysis of (A) caspase-3 and cleaved caspase-3, (B) β-actin, (C) GSDME and GSDME-N, and (D) β-actin in 4T1 cells under normoxia condition. From G1 to G6, they are Control, Laser, SIRPI, SIRPI+Laser, SIRPI+H_2_O_2_, SIRPI+Laser+H_2_O_2_, respectively.


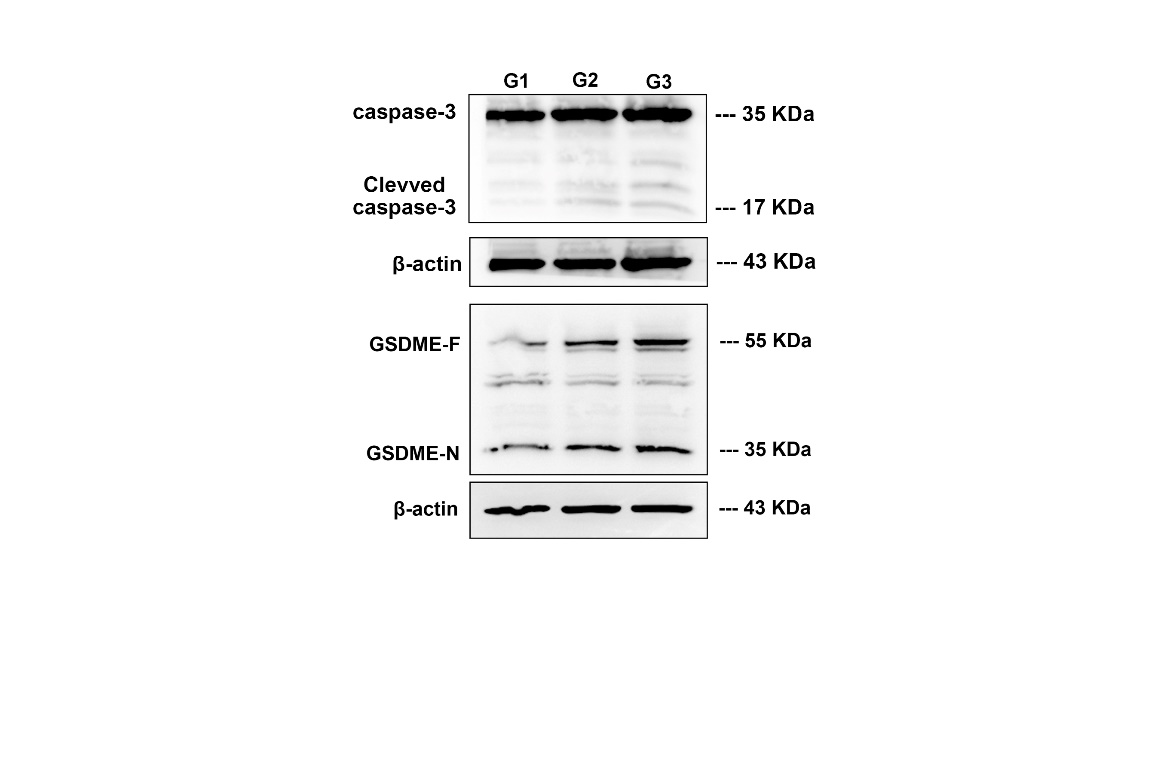


**Figure S17.** Expressions of cleaved caspase-3 and GSDME-N in MCF-7 cells using WB analysis. From G1 to G3, they are Control, SIRPI, SIRPI+Laser+H2O2, respectively.


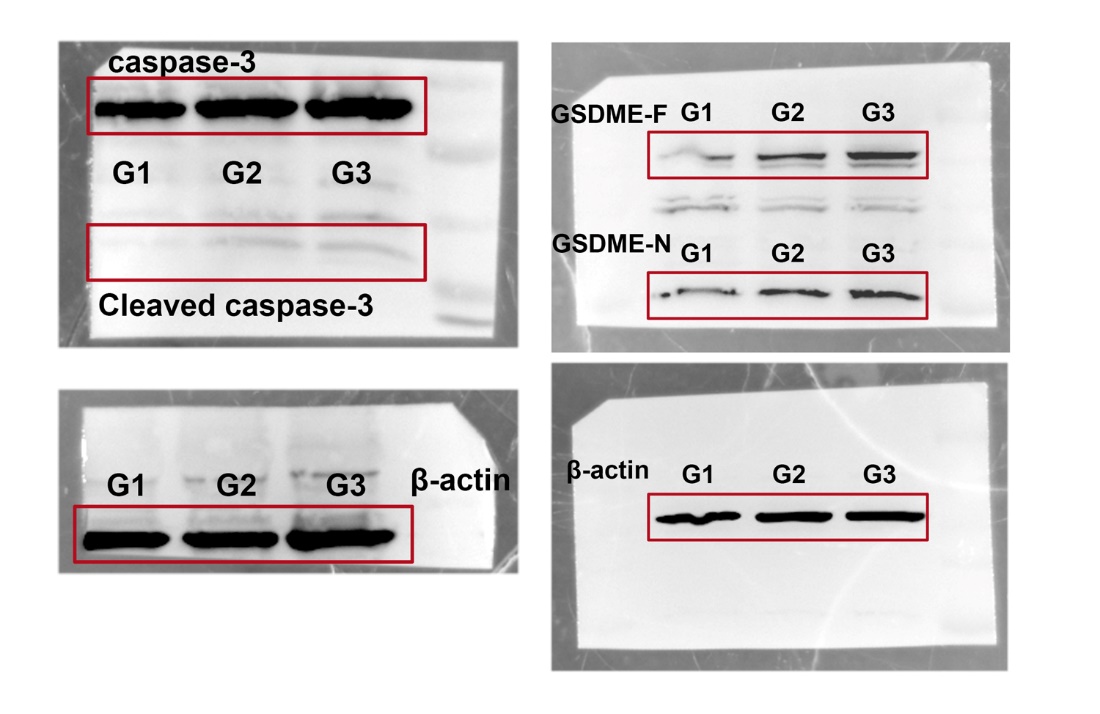


**Figure S18.** WB analysis of caspase-3, cleaved caspase-3, GSDME and GSDME-N in MCF-7 cells. From G1 to G3, they are Control, SIRPI, SIRPI+Laser+H_2_O_2_, respectively.


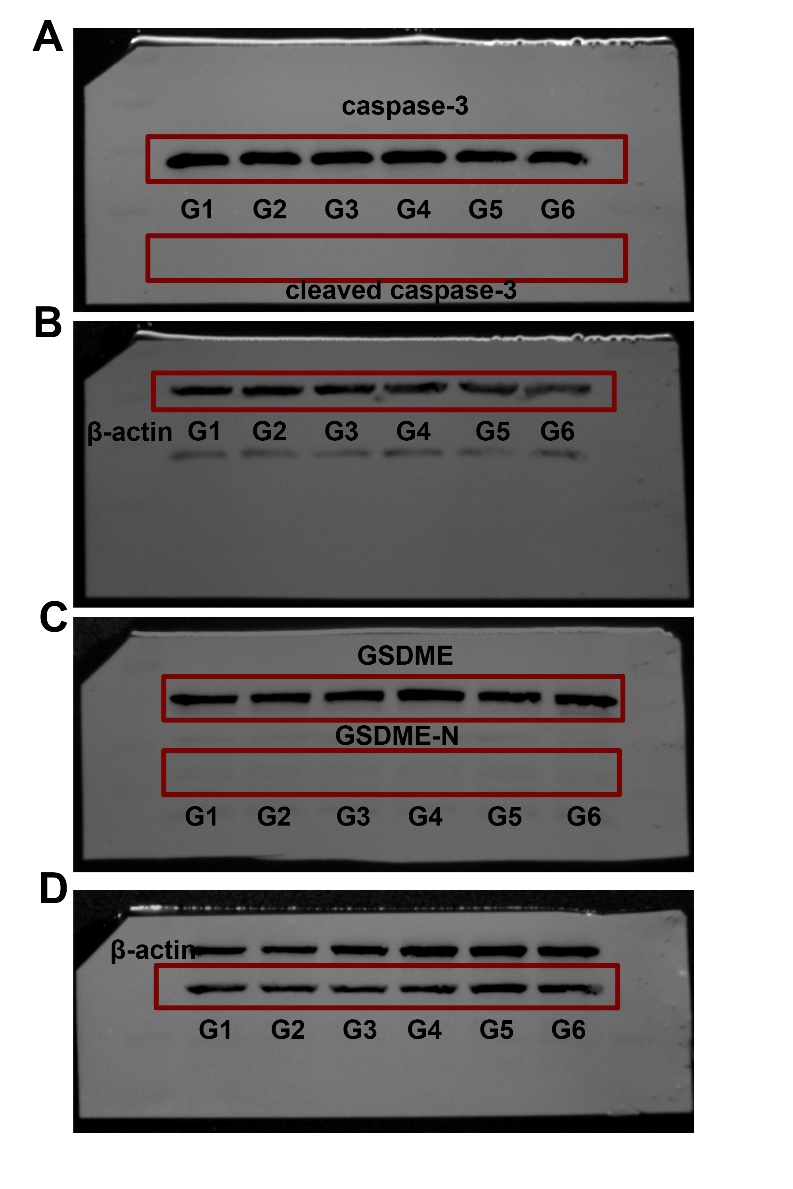


**Figure S19.** WB analysis of (A) caspase-3 and cleaved caspase-3, (B) β-actin, (C) GSDME and GSDME-N, and (D) β-actin in Hs578bst cells under normoxia condition. From G1 to G6, they are Control, Laser, SIRPI, SIRPI+Laser, SIRPI+H_2_O_2_, SIRPI+Laser+H_2_O_2_, respectively.


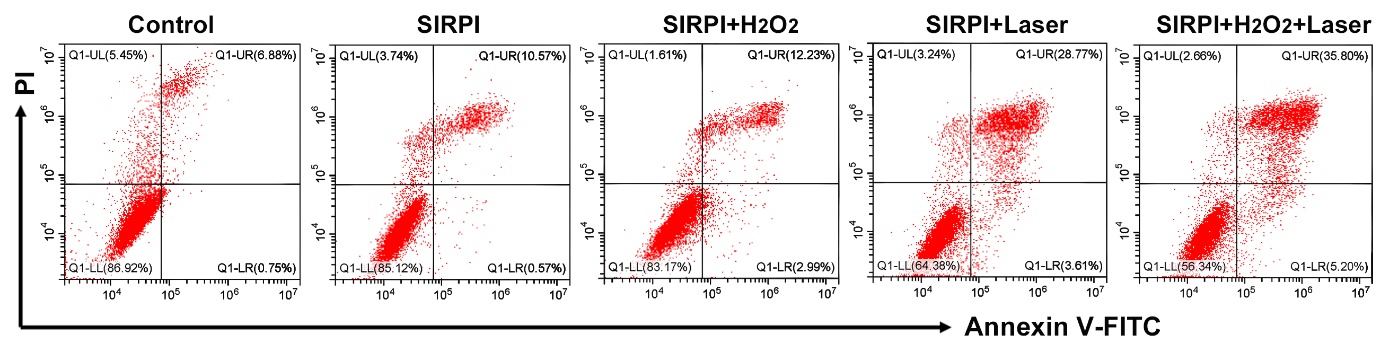


**Figure S20.** Flow cytometry analysis of the apoptosis of 4 T1 cells in different treatments.


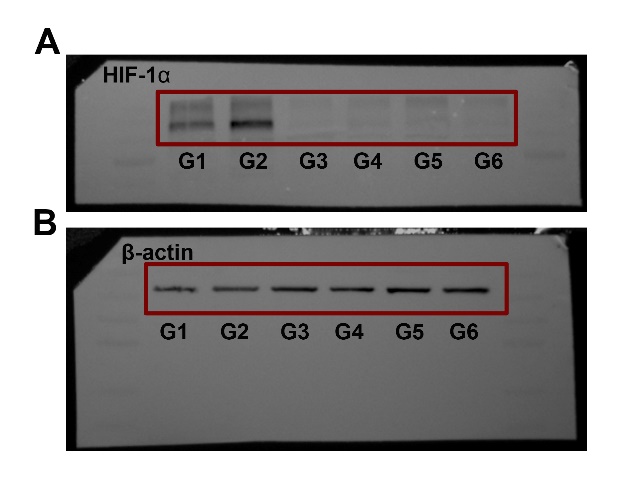


**Figure S21.** WB analysis of (A) HIF-1α and (B) β-actin 4T1 cells under hypoxia condition. From G1 to G6, they are Control, Laser, SIRPI, SIRPI+Laser, SIRPI+H_2_O_2_, SIRPI+Laser+H_2_O_2_, respectively.


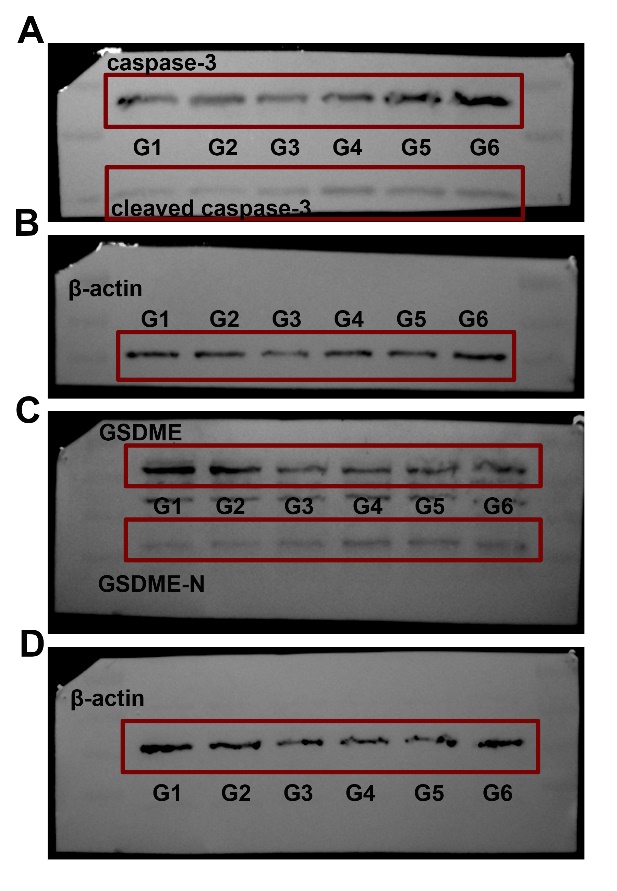


**Figure S22.** WB analysis of (A) caspase-3 and cleaved caspase-3, (B) β-actin, (C) GSDME and GSDME-N, and (D) β-actin in 4T1 cells under hypoxia condition. From G1 to G6, they are Control, Laser, SIRPI, SIRPI+Laser, SIRPI+H_2_O_2_, SIRPI+Laser+H_2_O_2_, respectively.


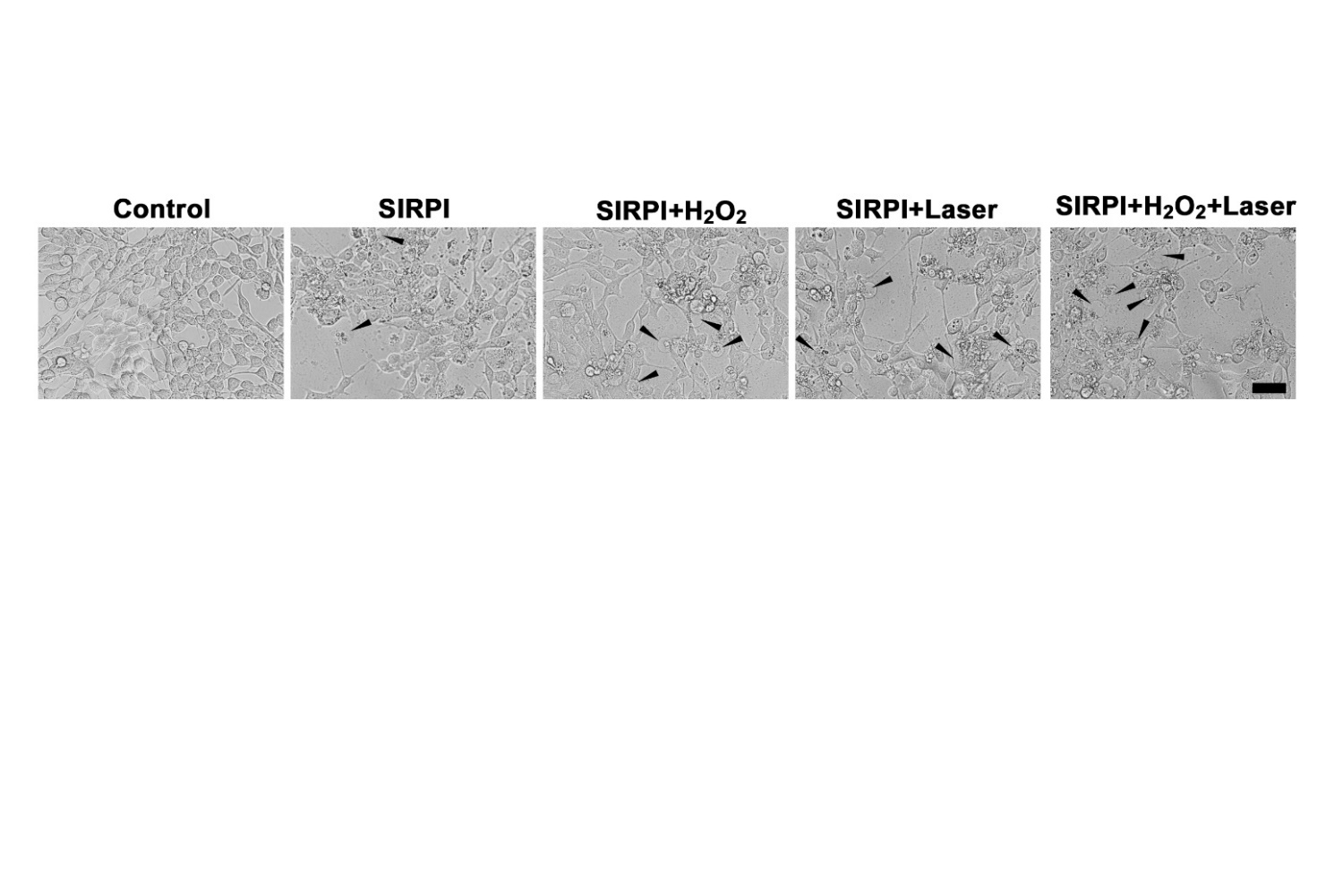


**Figure S23.** The morphology observation of 4T1 cells with different treatment under hypoxia condition, the scale bar is 100 μm.


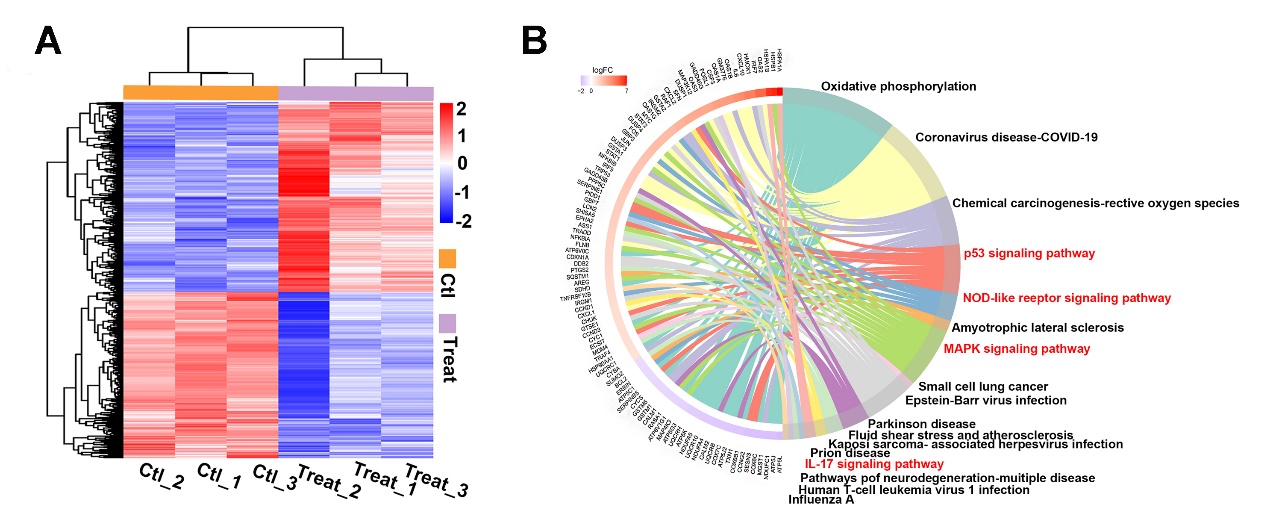


**Figure S24.** (A) Cluster diagram of DEGs between the control and SIRPI+Laser+H_2_O_2_ groups. The Ctl and Treat represent the control and SIRPI+Laser+H_2_O_2_ respectively. (B) The circle diagram of gene ontology (GO) enrichment analysis of oxidative stress and inflammation related DEGs.


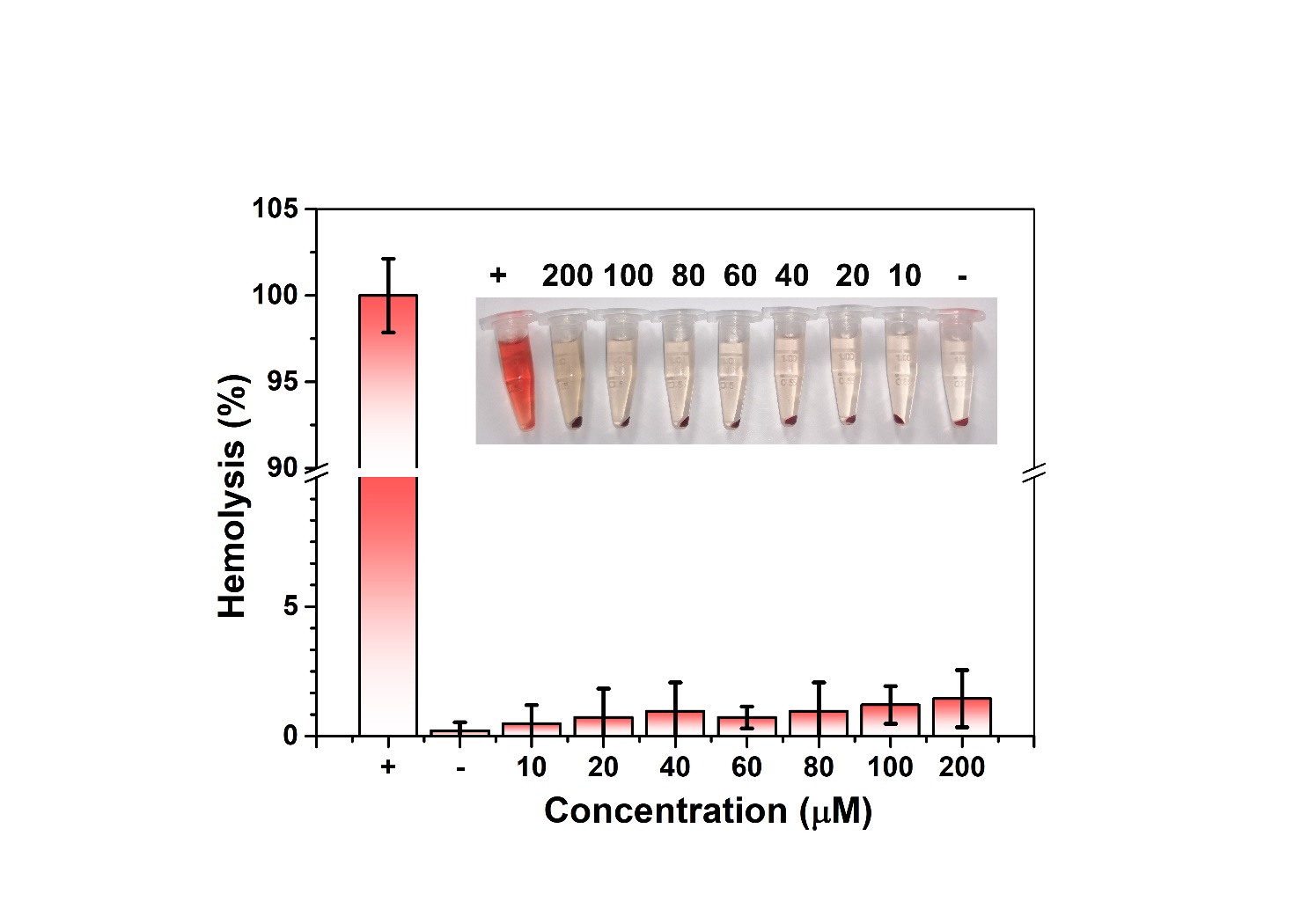


**Figure S25.** Hemolysis assays of water, PBS, and SIRPI at different concentrations (0, 10, 20, 40, 60, 80, 100, and 200 μM).

**
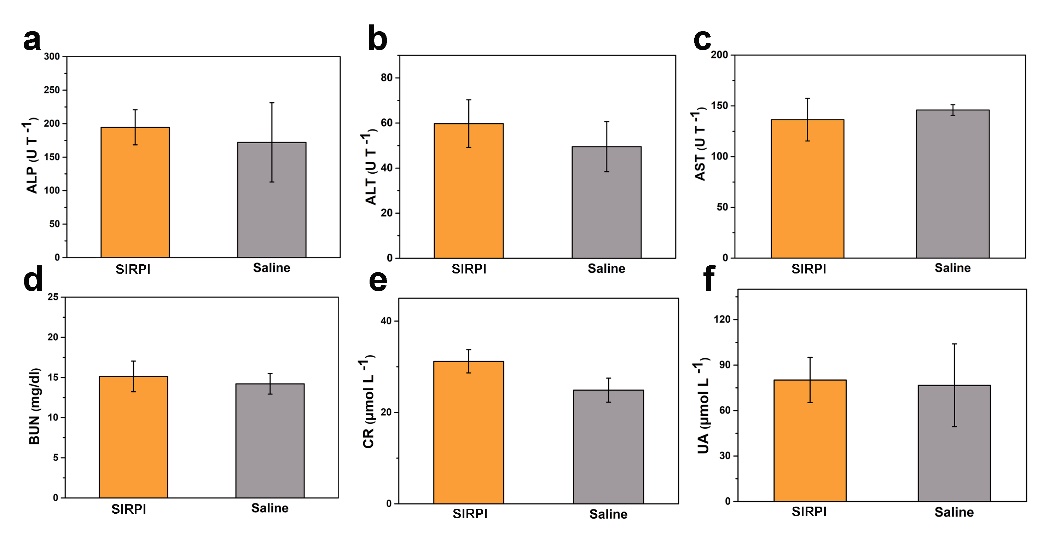
**

**Figure S26. Biocompatibility analyses of 6s on cellular level.** The biochemical indexes of (A) ALP, (B) ALT, (C) AST, (D) BUN, (E) CR, and (F) UA in the SIRPI and Saline treated mice.

**
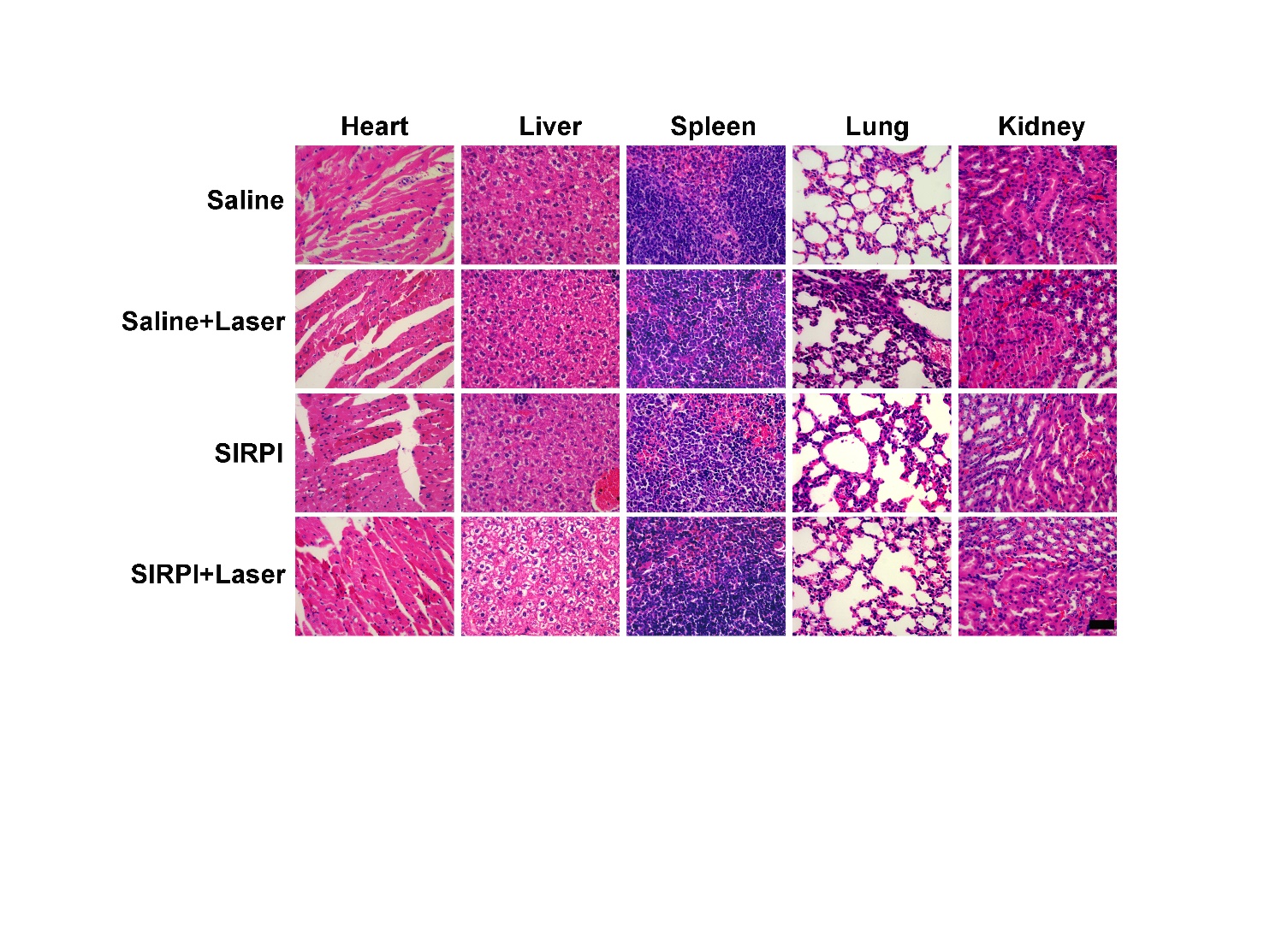
**

**Figure S27.** H&E staining images of heart, liver, spleen, lung, and kidney of the mice with different treatments, the scale bar is 50 μm.

**
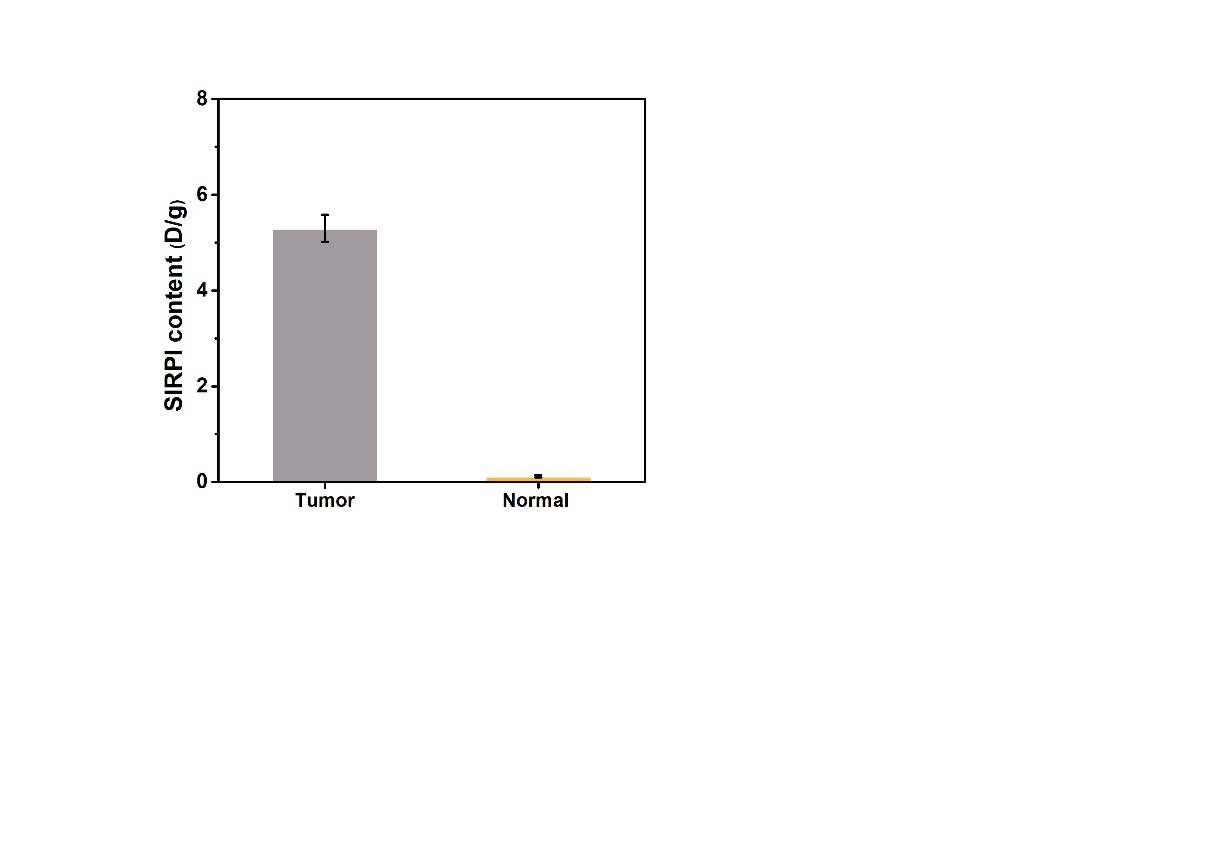
**

**Figure S28.** SIRPI enrichment in tumors and normal tissues after i.v. for 1 h.


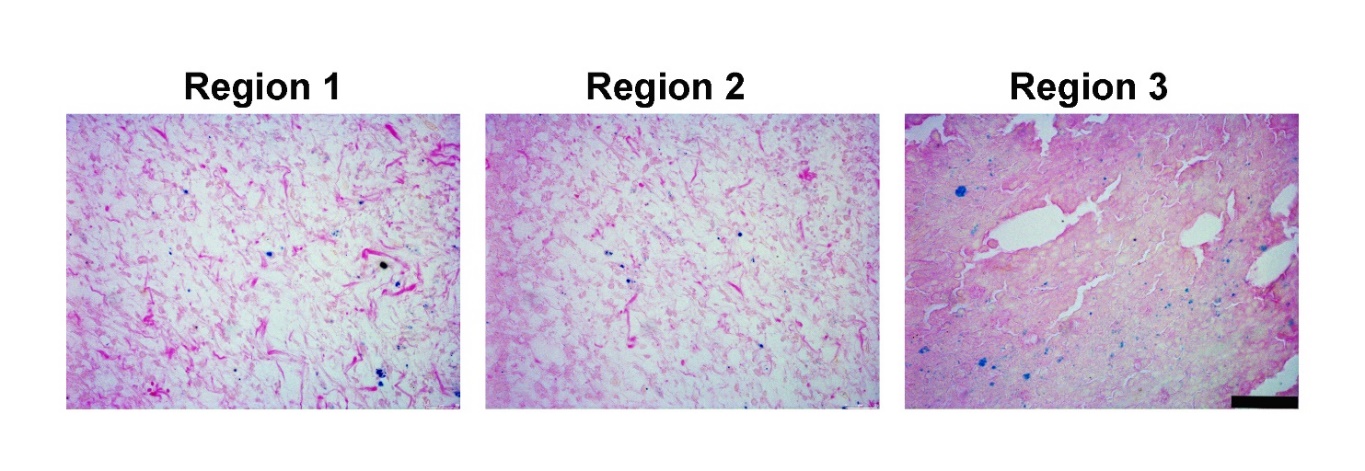


**Figure S29.** Prussian blue staining images of tumor to evaluate the enrichment of SIRPI in tumor tissues after i.v. for 1 h, the scale bar is 50 μm.

**
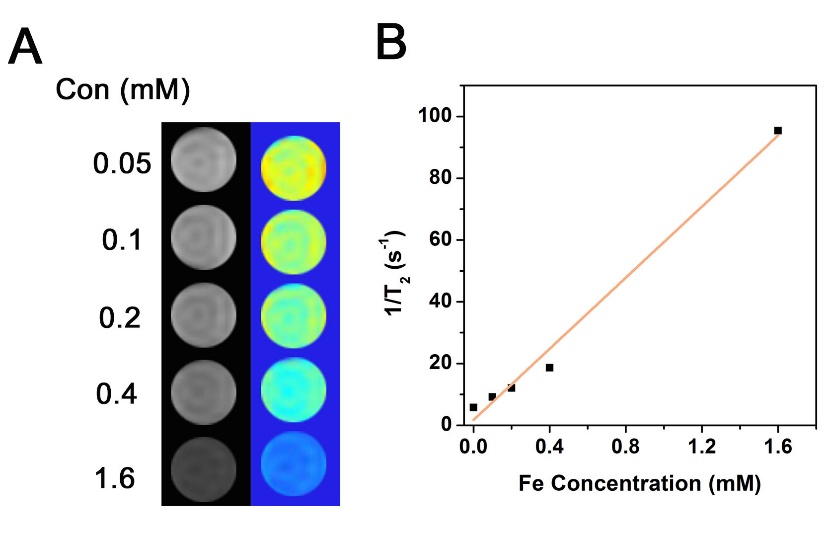
**

**Figure S30.** (A) *T*_2_ weighted images and magnified pseudocolor MR image of SIRPI. (B) The analyses of relaxation rate R_2_ (1/*T*_2_) Vs Fe concentration of SIRPI.


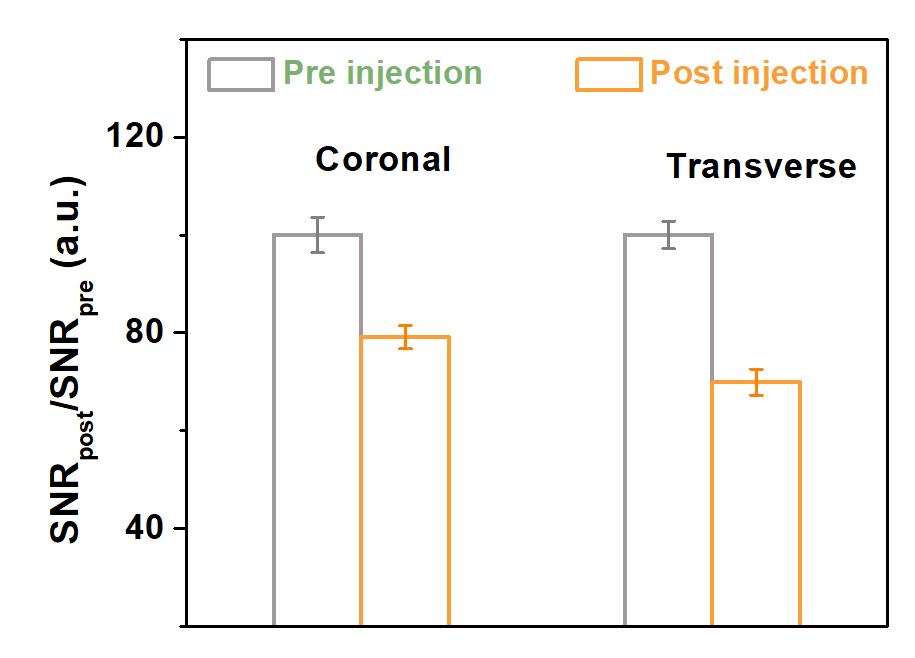


**Figure S31.** Quantification of SNR _post_/SNR _pre_ values in the transverse and coronal planes.

**
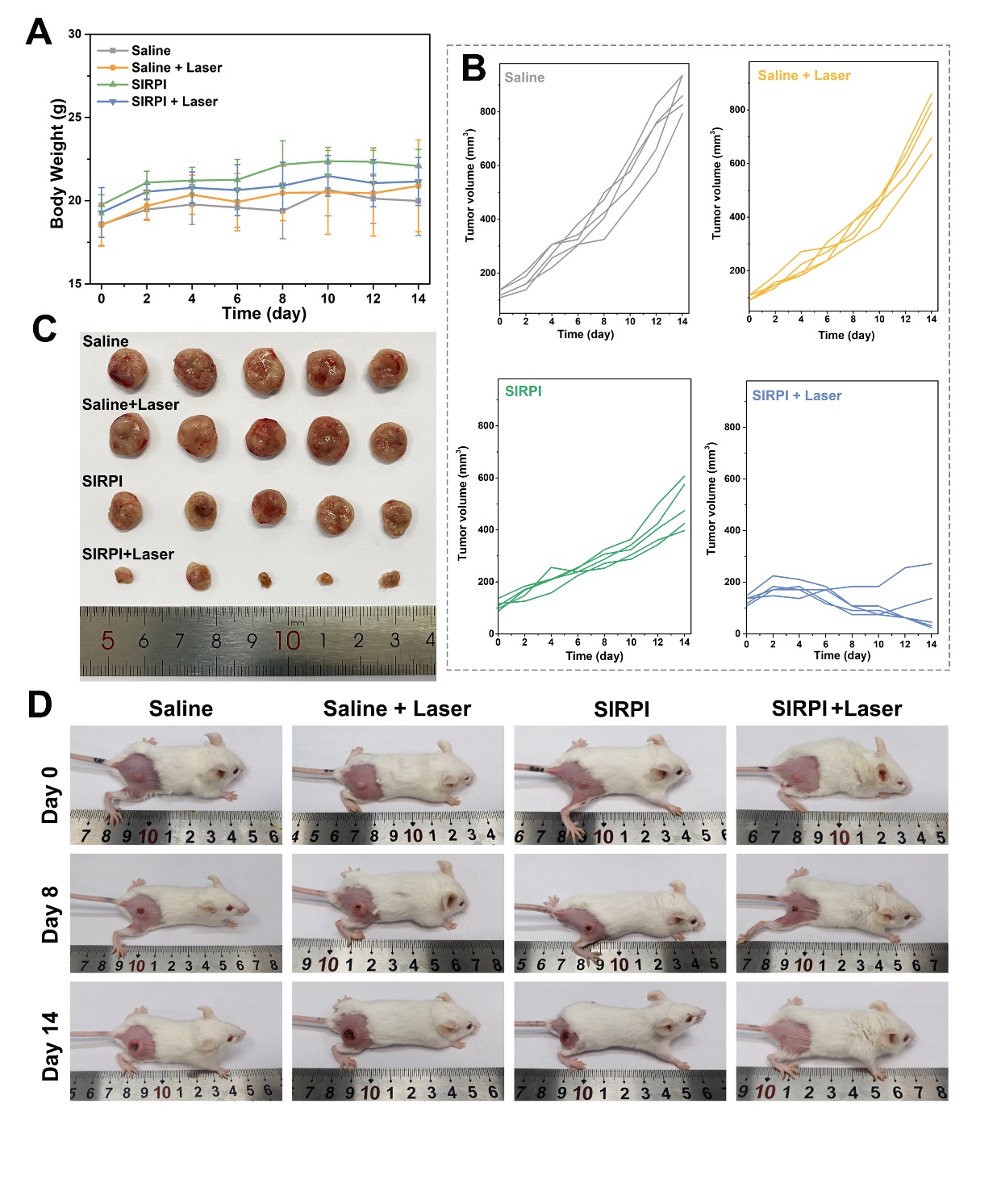
**

**Figure S32.** (A) Body weight changes, (B) individual tumor growth curves and (C) representative tumor images r of 4T1 tumor-bearing Balb/c mice with different treatments. (D) Tumor growth photograph of mice in different treatment groups within 14 days.


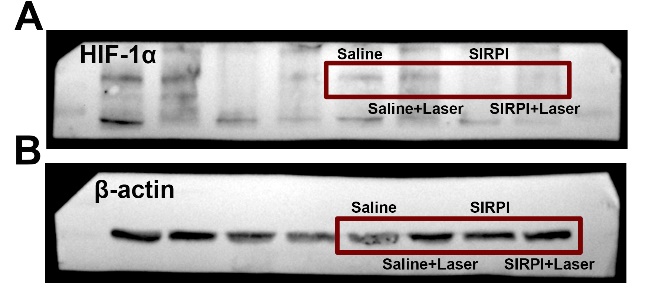


**Figure S33.** WB analysis of (A) HIF-1α and (B) β-actin in tumor tissues of 4T1 tumor-bearing Balb/c mice.


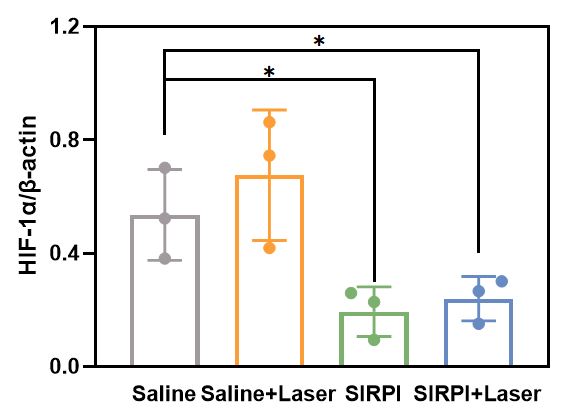


**Figure S34.** Quantitative analysis of HIF-1α in tumor tissues of 4T1 tumor-bearing Balb/c mice based on the WB results (*n* = 3).


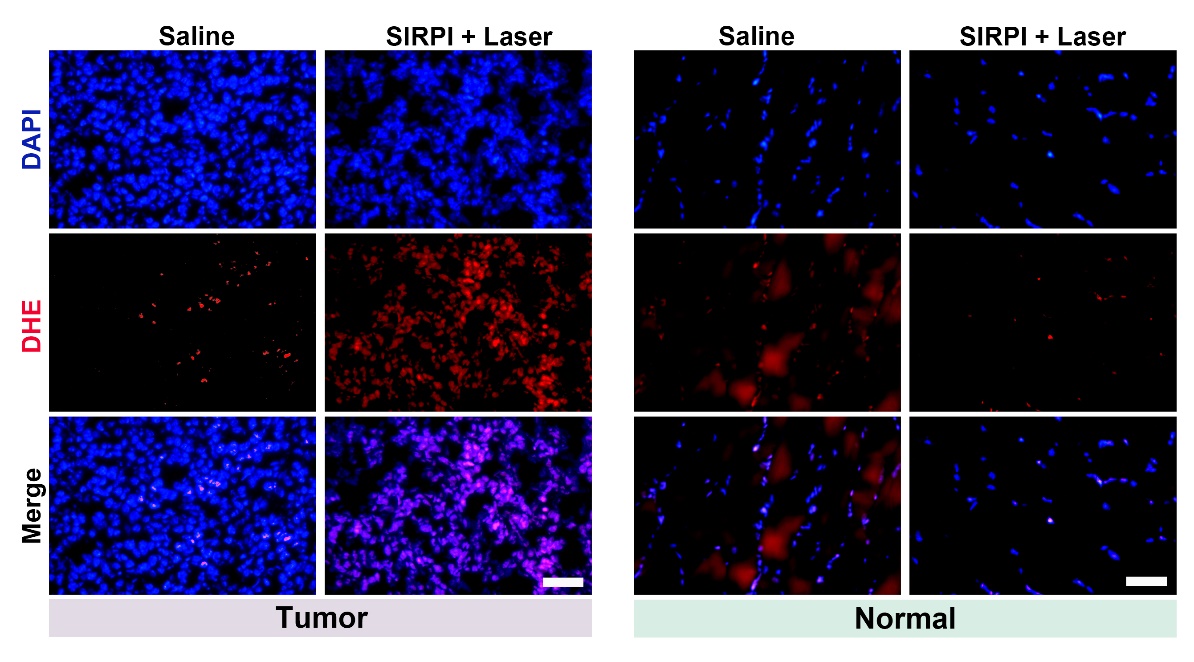


**Figure S35.** DHE staining of tumor and normal tissues from different treatments, scale bar is 50 μm.


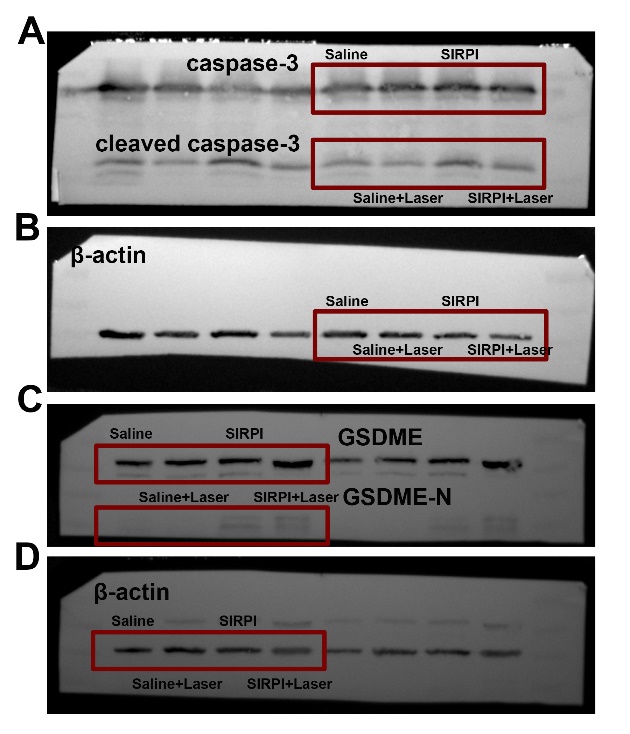


**Figure S36.** WB analysis of (A) caspase-3 and cleaved caspase-3, (B) β-actin, (C) GSDME and GSDME-N, and (D) β-actin in tumor tissues of 4T1 tumor-bearing Balb/c mice.


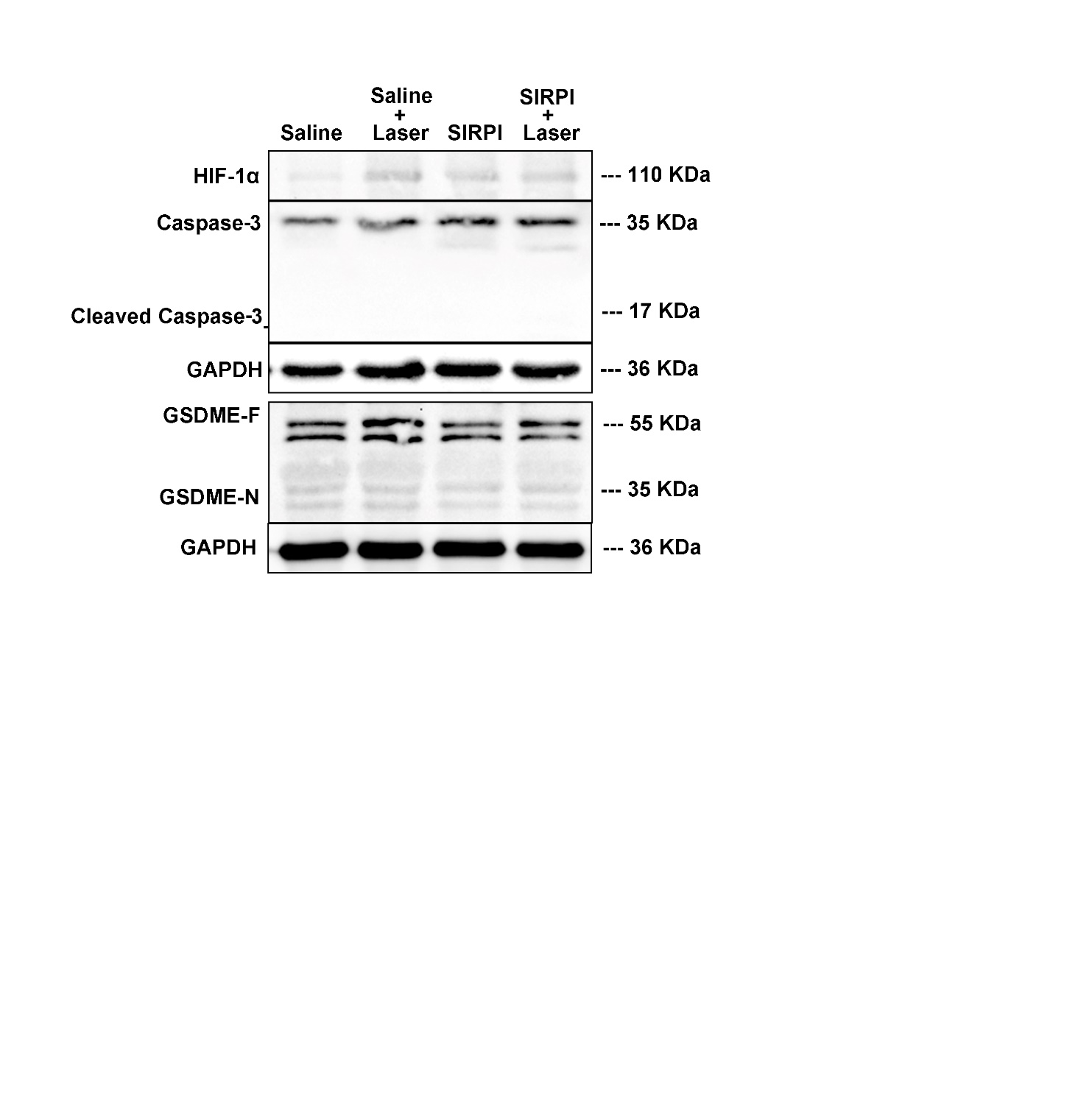


**Figure S37.** WB analysis of HIF-1α cleaved caspase-3 and GSDME-N in normal tissues of 4T1 tumor-bearing Balb/c mice after intravenous injection of SIRPI.


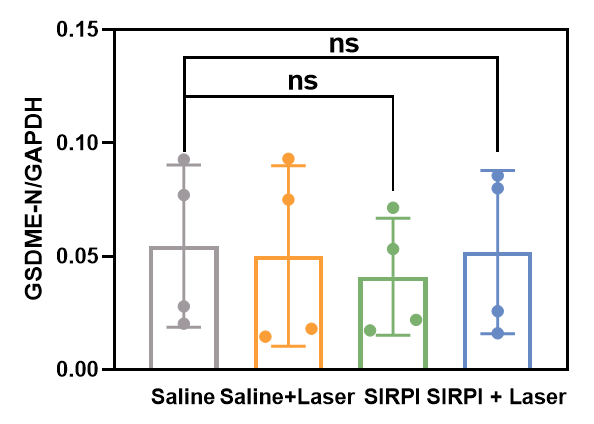


**Figure S38.** Quantitative analysis of GSDME-N in normal tissues of 4T1 tumor-bearing Balb/c mice after intravenous injection of SIRPI based on the WB results (*n* = 3).

**
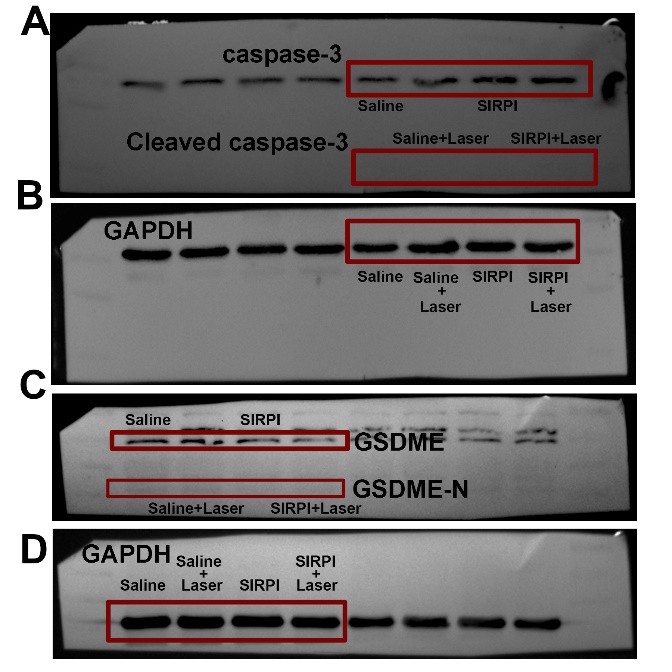
**

**Figure S39.** WB analysis of (A) caspase-3 and cleaved caspase-3, (B) GAPDH, (C) GSDME and GSDME-N, and (D) GAPDH in normal tissues of 4T1 tumor-bearing Balb/c mice (intravenous administration of SIRPI).

**
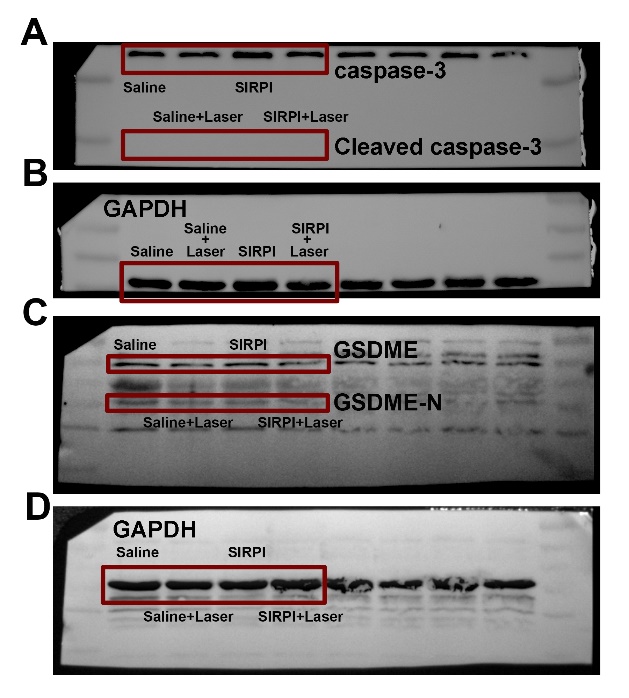
**

**Figure S40.** WB analysis of (A) caspase-3 and cleaved caspase-3, (B) GAPDH, (C) GSDME and GSDME-N, and (D) GAPDH in normal tissues of 4T1 tumor-bearing Balb/c mice (injected the same dose of SIRPI as enriched in tumor tissues using subcutaneous injections into normal tissues).


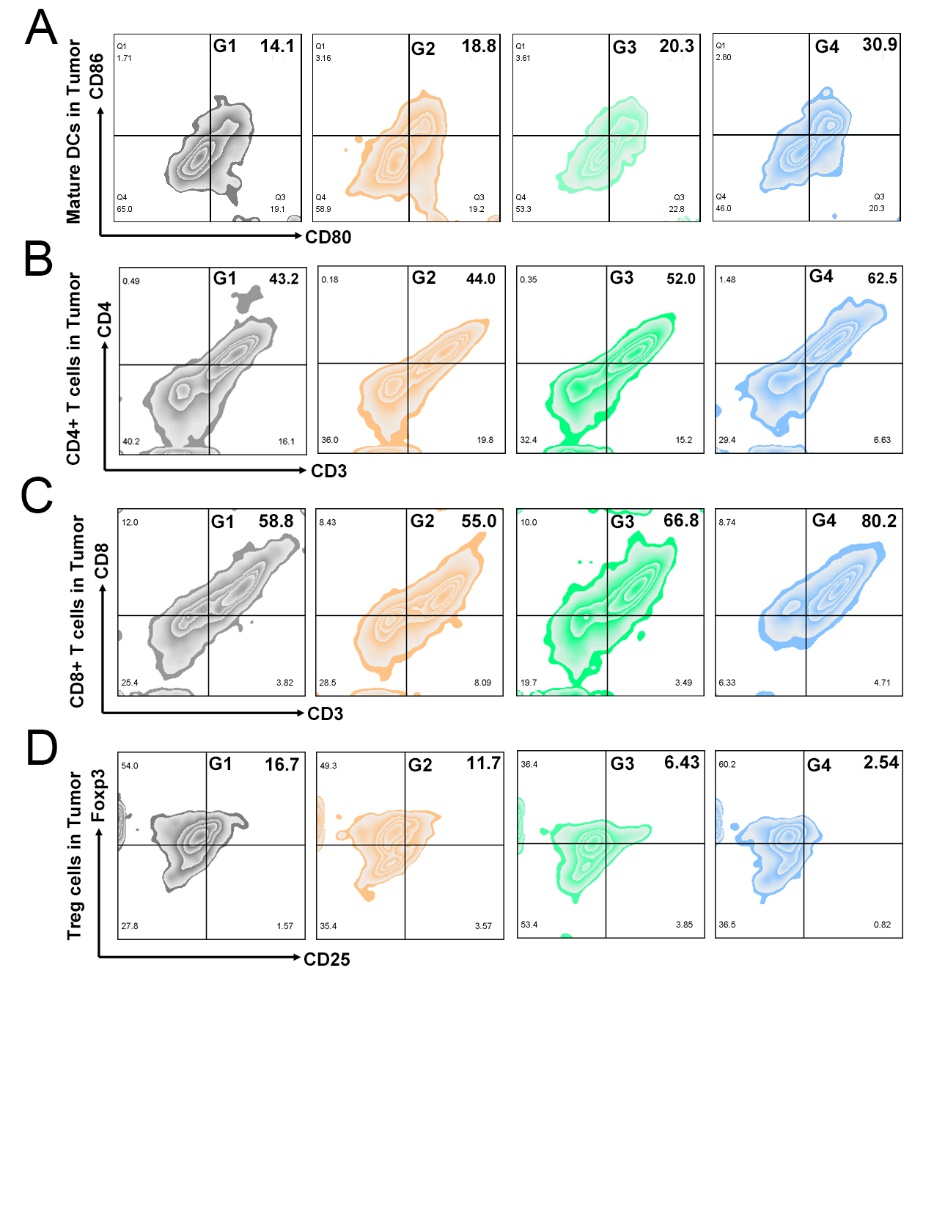


**Figure S41.** Flow cytometry analyses of (A) CD80^+^ CD86^+^ DCs, (B) CD4^+^ T cells, (C) CD8^+^ T cells, and (D) CD25^+^ Foxp3^+^ Tregs in the tumor.

**
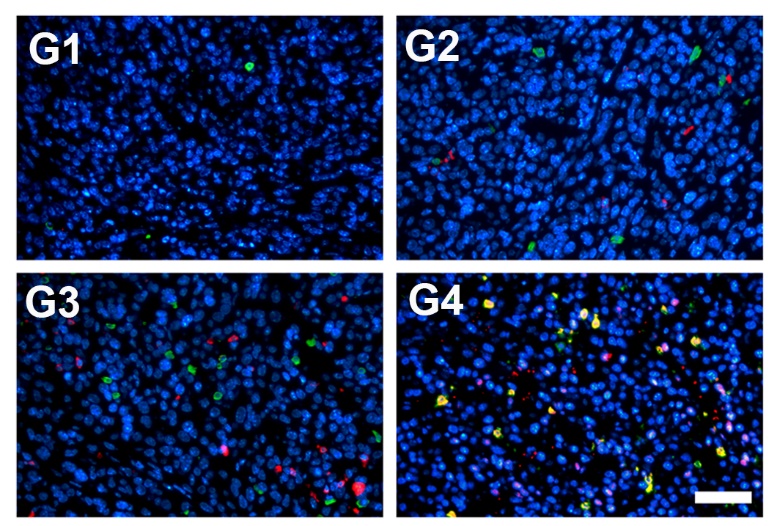
**

**Figure S42.** Representative immunohistochemical staining of CD4^+^ T cells and CD8^+^ T cells in tumor sections of each group, the scale bar is 50 μm. From G1 to G4, they are Saline, Saline+Laser, SIRPI, SIRPI+Laser, respectively.


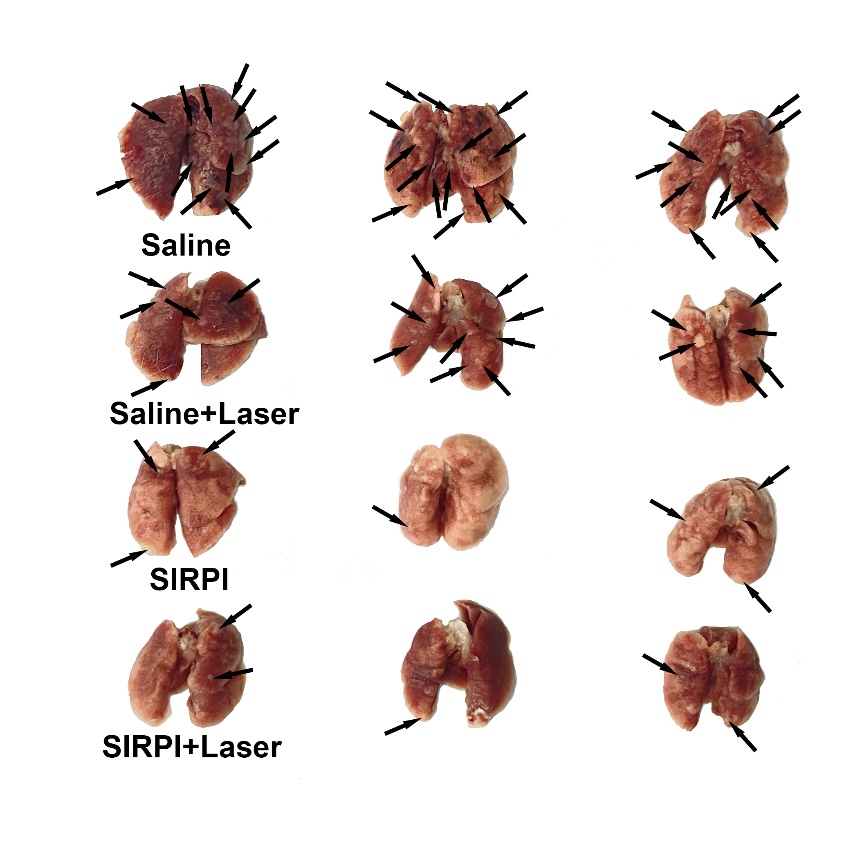


**Figure S43.** Representative image of lung metastatic tumors after treatments on day 23. The metastatic regions were marked by the black arrows.


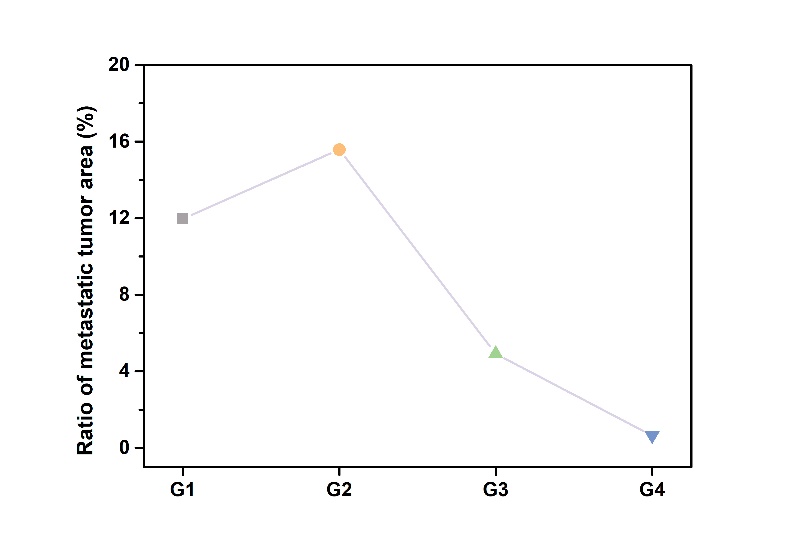


**Figure S44.** Calculated ratio of metastatic tumors areas in the total lung tissue areas in each group. From G1 to G4, they are Saline, Saline+Laser, SIRPI, SIRPI+Laser, respectively.
